# Efficacy of a multiple-component and multifactorial personalized fall prevention program in a mixed population of community-dwelling older adults with stroke, Parkinson's Disease, or frailty compared to usual care: The PRE.C.I.S.A. randomized controlled trial

**DOI:** 10.3389/fneur.2022.943918

**Published:** 2022-09-01

**Authors:** Fabio La Porta, Giada Lullini, Serena Caselli, Franco Valzania, Chiara Mussi, Claudio Tedeschi, Giulio Pioli, Massimo Bondavalli, Marco Bertolotti, Federico Banchelli, Roberto D'Amico, Roberto Vicini, Silvia Puglisi, Pierina Viviana Clerici, Lorenzo Chiari, The PRECISA Group, Fabio La Porta

**Affiliations:** ^1^IRCCS Istituto delle Scienze Neurologiche di Bologna, Bologna, Italy; ^2^Rehabilitation Unit, Azienda Ospedaliero-Universitaria di Modena, Modena, Italy; ^3^Azienda Ospedaliera Arcispedale Santa Maria Nuova, Reggio Emilia, Italy; ^4^Unit of Statistical and Methodological Support for Clinical Research, University of Modena and Reggio Emilia, Modena, Italy; ^5^Department of Electrical, Electronic, and Information Engineering “Guglielmo Marconi” (DEI), University of Bologna, Bologna, Italy; ^6^Health Sciences and Technologies Interdepartmental Center for Industrial Research (CIRI-SDV), University of Bologna, Bologna, Italy

**Keywords:** accidental falls, frail elderly, stroke, Parkinson's Disease, primary prevention, randomized controlled trial, independent living, rehabilitation

## Abstract

**Background:**

Fall risk in the elderly is a major public health issue due to the injury-related consequences and the risk of associated long-term disability. However, delivering preventive interventions in usual clinical practice still represents a challenge.

**Aim:**

To evaluate the efficacy of a multiple-component combined with a multifactorial personalized intervention in reducing fall rates in a mixed population of community-dwelling elderly compared to usual care.

**Design:**

Randomized Controlled Trial (NCT03592420, clinicalTrials.gov).

**Setting:**

Outpatients in two Italian centers.

**Population:**

403 community-dwelling elderly at moderate-to-high fall risk, including subjects with Parkinson's Disease and stroke.

**Methods:**

After the randomization, the described interventions were administered to the intervention group (*n* = 203). The control group (*n* = 200) received usual care and recommendations to minimize fall risk factors. In addition, each participant received a fall diary, followed by 12 monthly phone calls. The primary endpoint was the total number of falls in each group over 12 months, while the secondary endpoints were other fall-related indicators recorded at one year. In addition, participants' functioning was assessed at baseline (T1) and 3-month (T3).

**Results:**

690 falls were reported at 12 months, 48.8% in the intervention and 51.2% in the control group, with 1.66 (± 3.5) and 1.77 (± 3.2) mean falls per subject, respectively. Subjects with ≥ 1 fall and ≥2 falls were, respectively, 236 (58.6%) and 148 (36.7%). No statistically significant differences were observed between groups regarding the number of falls, the falling probability, and the time to the first fall. According to the subgroup analysis, no significant differences were reported. However, a statistically significant difference was found for the Mini-BESTest (*p* = 0.004) and the Fullerton Advanced Balance Scale (*p* = 0.006) for the intervention group, with a small effect size (Cohen's d 0.26 and 0.32, respectively), at T1 and T3 evaluations.

**Conclusions:**

The intervention was ineffective in reducing the number of falls, the falling probability, and the time to the first fall at 12 months in a mixed population of community-dwelling elderly. A significant improvement for two balance indicators was recorded in the intervention group. Future studies are needed to explore different effects of the proposed interventions to reduce falls and consequences.

## Introduction

Fall risk in the elderly is a major public health issue due to the immediate injury-related consequences and the risk of associated long-term disability (1). One out of three older people over 65 years is estimated to fall each year and this rate increases to 50% in the elderly over 80 (2). Furthermore, around 15% of older adults are multiple fallers, experiencing more than one fall each year, thus increasing morbidity and mortality (1). In 2 to 10% of cases, falls can lead to hip fractures related to functional decline, death, and increased hospitalization costs, even though falls alone limit, *per se*, social participation and may increase the risk of institutionalization (3). Moreover, the costs for the acute management of the 85,762 hospitalizations for hip fractures that occurred in Italy in 2005 were estimated to be around 467 million Euros, with rehabilitation costs reaching 532 million Euros in the same year (4). In Regione Emilia-Romagna (Italy), Berti and colleagues (5) reported 5,904 yearly hip fractures in 2017. Referring to a conceptual framework for a hip fracture integrated episode of care, defined as Continuum-Care Episode (CCE), they estimated a median cost of 7,404.5 euros for the acute phase and a median cost of 3,449.6 euros for the rehabilitative one. Therefore, an effective fall prevention intervention is of primary importance also to reduce this tremendous socioeconomic burden.

A systematic literature review and meta-analysis analyzed fall risk factors in community-dwelling older people (6), highlighting that falling results from an interaction between environmental hazards and inadequate physiology to cope with them, such as gait problems, poor vision, impaired peripheral sensation, lower limb strength, dizziness, and the use of psychotropic medications or polypharmacy (6, 7). Therefore, guidelines recommend a multifactorial fall risk assessment in older adults presenting for medical attention after a fall or who have gait or balance problems (8). This strategy implies identifying modifiable risk factors and implementing targeted interventions for fall prevention (3). However, the delivery of effective treatments for fall prevention in usual clinical practice still represents a challenge (9, 10).

A 2013 Cochrane review (1) on fall prevention for community-dwelling older people identified three effective interventions: single-component, multiple-component, and multifactorial. A single-component intervention consists of only one major intervention category, whereas the multiple-component one is a fixed combination of two or more major intervention categories. Typically, single- and multiple-component interventions are always delivered to all subjects. On the other hand, a multifactorial intervention consists of two or more interventions, which are delivered in different combinations to each individual based on a personalized assessment to identify potential risk factors for falling (1). This systematic review identified that multifactorial interventions reduced the rate of falls (i.e., the total number of falls per unit of person time that falls were monitored) but not the risk of falling (i.e., the risk ratio that compares the number of people who fell once or more). Only exercise (either delivered as a multiple-component group exercise or home-based exercise) and home safety interventions reduced both (1). Furthermore, another Cochrane systematic review issued in 2020 highlighted that exercise programs reduced the rate of falls and the number of people experiencing falls in older people living in the community (11). In particular, programs based on balance and strength training were particularly effective at preventing falls.

On the other hand, a recent systematic review by Lee and Yu (12) reported that ‘active' multifactorial interventions (i.e., that actively assessed risk factors and resolved fall-related problems) had significant positive effects both on fall rates and the number of people experiencing falls (12). These results contrast with those by Morello et al. (13), who highlighted the lack of sufficient evidence to support the use of multifactorial interventions to prevent falls or reduce hospital utilization in older people presenting to the Emergency Department following a fall (13). Furthermore, in an ongoing Randomized Control Trial (RCT) (14), multiple and multifactorial interventions were employed to prevent falls in community-dwelling older people. However, results on treatment effectiveness are not available yet. Besides, in 2020 Lamb et al. demonstrated that screening by mail followed by a targeted exercise intervention or multifactorial approach to preventing falls did not result in a lower rate of fractures than advice by mail alone (15). Finally, RCTs in the two cited Cochrane systematic reviews did not include subjects with neurological conditions, such as Parkinson's Disease (PD) and stroke. Therefore, there is conflicting evidence about the most effective interventions in reducing the rate of falls and the risk of falling in community-dwelling older adults.

It is worth noticing that all the cited studies excluded community-dwelling older adults with associated neurological conditions. On the other hand, evidence from the literature showed that among these subjects with neurological disorders, there is a high proportion of fallers with a high rate of participation restriction (16, 17). Previous studies suggested that exercise improves balance in PD, even though the fall rate and risk reduction were not achieved (18–20). A recent study (19) investigated a combination of educational and exercise interventions to reduce falls in people with neurological conditions: results from this RCT did not show a reduction in fall risk. However, to the best of the authors' knowledge, no studies were conducted on a combined intervention to prevent falls in the elderly living in the community, including participants affected by neurological conditions, and with a synergy between group exercise and personalized home exercise to increase compliance and chances that home exercise may become an integral part of a long-term more active and healthier lifestyle.

We hypothesized that the multiple-component intervention associated with a personalized multifactorial assessment and intervention delivered to the treatment group, composed of community-dwelling older adults with or without an associated neurological disease (i.e., stroke or PD), would be more effective than usual care at reducing the number of falls and the falling probability, as well as delaying the time to the first fall, at a twelve-month follow-up. Furthermore, we also hypothesized that the synergy between group exercise and personalized home exercise proposed in the trial would increase compliance and the routinary integration of home exercise in the older adults' long-term active and healthy lifestyle.

Thus, the current study aimed to evaluate the efficacy of a multiple-component intervention associated with a personalized multifactorial intervention to reduce fall rates in community-dwelling older adults who can walk but are at risk of falling, including those with PD and stroke, compared to usual care.

## Materials and methods

### Study design

**Figure 1:**
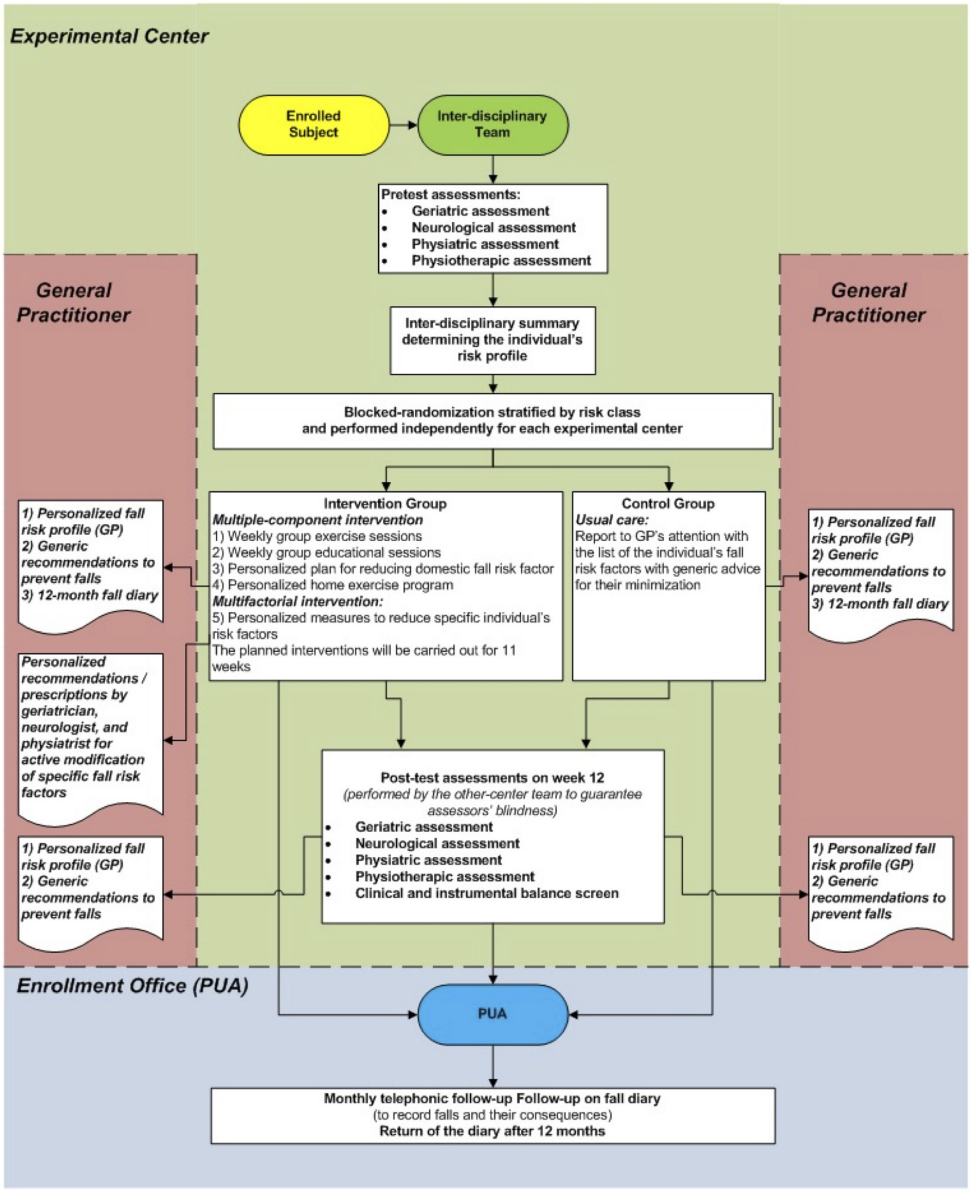
Study summary flow chart.

This study was a multicenter randomized controlled trial where individuals randomized to the intervention group (IG) received an 11-week multiple-component and personalized multifactorial intervention to reduce fall risk. In contrast, the control group (CG) participants received only usual care. Pre-test and post-test assessments were conducted, respectively, before randomization and 12 weeks after the commencement of the intervention. Primary and secondary endpoints were assessed at a twelve-month follow-up. The study design is presented in Figure 1.

The study was conducted in two Italian Public Hospitals (Ospedale Civile di Baggiovara in Modena and Arcispedale Santa Maria Nuova in Reggio Emilia) between 2015 and 2016. It was registered on clinicaltrials.gov (unique identifier NCT03592420) and approved by the local Ethical Committee (Provincial Ethics Committee of Modena 1141/CE/2014). Furthermore, all participants gave written informed consent to participate in the study, whose conduction adhered strictly to the Helsinki Declaration's ethical principles (21).

### Participants

Inclusion criteria were: age ≥65 years, moderate-to-high fall risk associated with age and/or neurological conditions (i.e., PD and stroke), ability to walk for at least 10 meters without assistance (possible use of a walking aid), and the agreement to give written informed consent to the study. The inclusion criteria “moderate-to-high fall risk” and “ability to walk for at least 10 meters without assistance” were assessed during the Anamnestic and the Objective Assessments of Eligibility for the enrolment through the administration of validated tests as described below.

Exclusion criteria were: general health conditions likely to interfere with or to pose a contraindication to physical exercise (e.g., severe heart disease, severe chronic artery disease, chronic respiratory problems, recent lower limbs fractures limiting weight bearing, etc.), cognitive impairment (Mini-Mental Test score <24 or cognitive conditions interfering with test administration), severe deafness, severe vision impairment, severe aphasia or visual-spatial disorders, subjective and objective vertigo in the last 3 months, ongoing physiotherapy likely to influence the target variables (at the time of enrolment).

### Enrolment algorithm

Health professionals (medical specialists or general practitioners) could signal to an enrolment office shared between the two centers (‘Punto Unico di Arruolamento', PUA) through an *ad hoc* case report any subjects over 65 years old, with or without a diagnosis of PD or stroke, considered to be “at fall risk”. The subject's compliance with the inclusion and exclusion criteria was declared in this form. Also, subjects considering themselves at risk of falling could self-report themselves through a dedicated email address.

After the initial report, potentially eligible participants were screened for inclusion and exclusion criteria in more detail. In particular, eligible subjects underwent further assessments by these subsequential steps:

1. Anamnestic Assessment of Eligibility (AAE):
  - This assessment was the PRE.C.I.S.A.'s first selection step. It was administered by a PUA's trained nurse, through a telephone call, to older adults who had been signaled as “at fall risk”;
  - The aim was to confirm the inclusion/exclusion criteria for the study recruitment and evaluate the most influential fall risk factors. In particular, the PUA's nurse verified the subjects' adherence to inclusion and exclusion criteria. Furthermore, the subjects were submitted to the Fall Risk Assessment Tool [FRAT, (22)] and were asked if they were afraid to fall and if they were able to walk 10 meters without assistance;
  - Later, each subject was classified as “not eligible and/or at low risk”, “moderate”, or “high” fall risk according to the defined algorithm indicated in Table 1;
  - Those who resulted at “low fall risk” or “not satisfying study criteria” were excluded. However, the study protocol allowed the PUA's nurse to contact these subjects after 1 year to record any eventual fall, thus providing helpful quantitative information for the *post-hoc* validation of the screening algorithm.
  - After combining the assessment results, people classified as being at “moderate-to-high fall risk” accessed the successive selection step (Objective Assessment of Eligibility - OAE).
  - The estimated time to perform this step was 5–10 min.
2. Objective Assessment of Eligibility (OAE):
  - This assessment constituted the PRE.C.I.S.A.'s. second selection step, and it was administered during an outpatient visit by a trained physiotherapist to individuals selected at “moderate-to-high fall risk” during the previous selection step (AAE);
  - The aim was to evaluate in detail all fall risk factors described in the literature and, hence, to confirm the eligibility for the study (be at “moderate-to-high fall risk” after the combination of the assessment results). In particular, the prospective participant was submitted to the Falls Risk for Older People in the Community Screen (FROP-Com Screen) (23), to the Fall Risk Assessment Tool [FRAT, (24)], to several tests for mobility and balance [Ten Meters Walking Test (25), Timed Up&Go test (26), Tandem stance from the 4 Stage Balance Test (27), 30-second Chair Standing test (28), Short Physical Performance Battery (29), Functional Reach Test (30)], to the Abbreviated Mental Test Score (31), and the visual acuity assessment (Snellen Chart);
  - Those who obtained an “eligible coefficient” ≥1, calculated from the FROP-Com Screen and the FRAT (24) total scores, were judged as “eligible” to be enrolled in the study (Table 1);
  - Any individual re-classified at “low risk” following this second assessment step was excluded from the enrolment and contacted 1 year later by the PUA's trained nurse to collect the number of any eventual falls that occurred.
  - The estimated time to perform this step was 20–30 min.

**Table 1:** AAE and OAE enrolment algorithms.

**Anamnestic Assessment of Eligibility (AAE) algorithm**	**Objective Assessment of Eligibility (OAE) algorithm**
*Not eligible* and/or at low risk	FROP-Com Screen
• the absence of at least one inclusion criteria OR • the presence of at least one exclusion criteria OR the inability to walk 10 meters without assistance OR • the ability to walk 10 meters without assistance AND a FRAT (22) total score equal to 0 AND the absence of fear of falling.	• Total score 0-1: low risk (eligible coefficient = 0) • Total score 2-4: medium risk (eligible coefficient = 1) • Total score 5-9: high risk (eligible coefficient = 2) FRAT (24) • Total score 5-11: low risk (eligible coefficient = 0)
*Moderate risk* • the ability to walk 10 meters without assistance AND the absence of previous fall(s), AND	• Total score 12-15: medium risk (eligible coefficient = 1) • Total score 16-20: high risk (eligible coefficient = 2).
• a FRAT (22) total score ≤ 2 OR • the presence of fear of falling.	
*High risk*	
• the ability to walk 10 meters without assistance AND • the presence of previous fall(s) OR • a FRAT (22) total score ≥3.	

### Randomization

After the enrolment, subjects judged as “eligible” participants underwent the pre-test assessments, which were conducted by a Physiatrist (P), a Physiotherapist (PT), a Geriatrician (G), and a Neurologist (N), as described in the following outcome measures section. These assessments helped determine in detail the individual fall risk profile.

Subsequently, the last assessor, who performed the pre-test evaluation, randomized the enrolled subject to the intervention group (IG) or the control group (CG). A web-based database was developed *ad hoc* for this study, and computer-based randomization was implemented to guarantee the allocation concealment. The randomization sequence was created using random block sizes of 4 and stratified according to risk classes, which distinguish subjects based on the different fall risks attributed in the literature depending on age and the presence of associated neurological diseases [older adults 65–79 years without associated neurological conditions: 33% (1); older adults ≥80 years without associated neurological disease: 50% (2, 32); older adults with stroke: 70% (33); older adults with PD: 60% (18)]. Participants were also stratified independently for each center.

After the randomization, all enrolled subjects were informed about the allocation arm and received a ‘usual care' intervention (Figure 1) based on:

- a report on their personalized risk factor profile;
- an illustrated brochure on fall prevention;
- personalized suggestions to minimize the fall risk addressed to their General Practitioner (GP).

Furthermore, all participants were provided with a one-year fall report diary, integrated with a physical activity monitoring diary, and several copies of a “fall report” that had to be filled by the participant, in case of a fall, with more detailed information about the event.

### Interventions

Participants in the IG were taken in charge by an interdisciplinary team, including the four professionals mentioned above, who administered synergically the following five interventions (described in detail in Supplementary material 1). In particular, a Physiatrist, two Physiotherapists (PTs), a Geriatrician, and a Neurologist were involved in each center. In addition, the team of four PTs designed their interventions and performed two training sessions together to minimize any possible inter-rater disagreements.

#### Group exercise sessions

This intervention consisted of progressive balance and strengthening training and dynamic balance and walking exercises, specifically for the risk class. Indeed, as already highlighted, two Cochrane systematic reviews showed that programs that challenge balance and use a higher dose of exercises have higher relative effects on reducing the rate of falls and the risk of falling (1, 11).

The intervention was based on 11 weekly group sessions (including six participants) for 60 min. Each session was composed of the three following parts:

- Warming-up (5 min): head, neck, trunk, and ankle movements, back and knee extensions, walking on the spot;
- Three-station circuit training (35 min): muscular strength exercises, balance exercises, and recovery techniques from falling (1);
- Dynamic balance and walking exercises specific for the risk class (10 min).

The remaining 10 min were used to check the physical activity report diary.

In the first session, the PT delivered a weight vest to each IG participant and verified the initial level for each of the three circuit training stations cited above. The personalization of the exercise intervention regarded the three-station circuit training (recovery techniques from falling and muscular strength and balance exercises), which were adapted individually. On the other hand, activities within the last part (dynamic balance and walking exercises) were not personalized but were adapted for the subject's risk class (older adults; older adults with stroke; older adults with PD). Further details are available in Supplementary material 1.

At each group session, the participants needed to have their weight vest, their fall-physical activity report diary of the current month, and their manual for the home exercise program (see next point 4). During rest periods from exercises, the participants delivered their fall-physical activity report diary and the completed 'fall report' in case of at least one fall during the week. At the end of the session, the PT updated the manual of the home exercise program with the week-level progression of the exercises (the passage changed every two weeks, but depending on individual need, it was possible to add other series of the same exercise in the intermediate weeks).

#### Group education sessions on fall risk factors

The IG participants received a thirty-minute educational session after each weekly group exercise session, focusing on different modifiable fall risk factors and avoidable risky behavior. The sessions were provided using appropriate language for lay elderly people and audio-visual material. The rationale of this intervention was to allow participants and their caregivers to identify everyday experiences and attitudes about the specific topic and to plan strategies to implement suggested prevention strategies for fall risk factors. Similar educational sessions had already been proposed in the study protocol of a Brazilian RCT (Prevquedas Brazil) on the effectiveness of a multifactorial fall prevention program in community-dwelling older people (14).

Each educational session was divided into two parts:

- A 10 min frontal lecture on a specific theme held by a component of the interdisciplinary team;
- A 20 min group discussion on the lecture content (involving participants, caregivers, and professionals). A handbook summarizing these topics was provided to each participant at the beginning of the first education session.

#### Personalized plan for reducing domestic fall risk factors

This intervention aimed at reducing domestic fall risk factors by their identification with an *ad hoc* questionnaire and the subsequent suggestions for their reduction given by a physiotherapist within four home visits. The rationale for a personalized plan for reducing domestic fall risk factors is that this intervention proved effective in reducing the rate of falls and the risk of falling in community-dwelling older adults, especially those at higher risk of falling, e.g., elderly with severe visual impairment (1).

During the first week of treatment, a PT performed a home visit for each IG participant. During this visit, usually lasting 60–90 min, the PT:

- Filled the “Home environmental risks questionnaire” and compared it with the same questionnaire compiled by the participant at the pre-test assessment;
- Gave specific recommendations with proposals for correcting the detected modifiable risk factors by delivering the “Suggestions for the reduction of environmental risks at home” information sheet where the actual hazards were highlighted;
- Verified the presence of the fall-physical activity report diary, delivered at the time of recruitment, in a position that facilitated its compilation in case of fall (e.g., hanging on the wall in the living room/ kitchen, etc.);
- During the subsequent three home visits related to the personalized home exercise program, the PT checked the implementation of the recommended interventions proposed during the first home access and filled the “Check-list of correction of environmental risk factors at home”.

#### Personalized home exercise program

This intervention was coordinated with the group exercise program aimed at improving strength, and static and dynamic balance, with the specific aim of enabling the participants to develop a long-term daily habit of exercising and performing physical activity in the context of a progressive and permanent adoption of a healthy and active lifestyle. The rationale for a home-based personalized exercise program is that the latter is known to be effective at reducing the rate of falls and the risk of falling in this population (1). Furthermore, the coordination of the home-based exercise protocol with the group-exercise program has already been proposed in the Prevquedas Brazil RCT study protocol (14).

The personalized home exercise program was realized according to the following steps:

- The PT devised this program in the context of an initial home visit (on the second week) and subsequently monitored within two further home visits (on the fourth and sixth weeks).
- During the initial home visit, an illustrated manual containing strength and balance exercises was provided and explained to each participant based on the first group exercise session. These exercises were chosen between those the subject performed with greater safety in the group session.
- Besides, the PT gave indications about recommended training frequency, as well as regarding time and registration of the performed physical activity in the fall-physical activity report diary of the current month.
- During the subsequent two visits, the PT verified: a) the setting adequacy, b) the modality in which the participant performed the suggested exercises, and c) the update of the fall-physical activity report diary.
- Finally, in all three home accesses linked to the home exercise program, the PT checked the implementation/maintenance of the recommendations on risk factors correction given in the first-week home visit.

#### Multifactorial personalized intervention

The rationale for this intervention is that a personalized multifactorial intervention is a well-known effective strategy to reduce both the fall rates and the risk of falling in elderly persons, as suggested by the already mentioned 2012 Cochrane systematic review (1) and by the 2020 systematic review by Lee and Yu (12), respectively. Furthermore, the multifactorial personalized intervention was included within the interventions carried out in the already mentioned Prevquedas Brazil RCT study protocol (14). This intervention, which was carried out by a multidisciplinary team, included the following actions:

- Review of medications, including psychotropic medications (N and G), antiparkinsonian drugs (N), and cardiovascular medications (G);
- Management of unaddressed visual impairments (G): ophthalmologist referral, lens prescription, suggestions regarding the limitation of bifocal lenses;
- Management of unaddressed cardiovascular issues (G), such as postural hypotension, covert cardiac failure, and abnormalities of cardiac rhythm; eventual cardiology referral;
- Vitamin D prescription (G);
- Improvement of nutritional state (G), with prescription of caloric-proteic integration and/or nutritional referral;
- Management of muscle-skeletal issues, including spasticity (P and PT);
- Education about foot self-care, including podologist referral if appropriate (P);
- Assessment, prescription, and final testing of orthosis and mobility aids, including proper shoes (P and PT).

#### Interventions delivery

Interventions one to four were administered to all IG participants (multiple-component intervention). In contrast, the multifactorial intervention (intervention number five) was personalized based on the individual fall risk profile devised on the pre-test assessment. Furthermore, interventions one, two, and five were conducted within an outpatient setting, while interventions three and four were home-based.

### Comparator

Participants allocated to the CG received only the usual care, as described in the randomization section. The fall risk management of each individual enrolled in the CG was delegated to the participant's GP.

### Outcome Measures

Participants' demographic and clinical characteristics were collected during the baseline pre-test visit, including age, sex, fall risk according to epidemiological criteria, and Falls Risk for Older People in the Community (FROP-Com) criteria.

Furthermore, several indicators were used by the interdisciplinary team to assess functioning at pre-test (T1) and three-month follow-up (T3). In addition, even instruments administered at the OAE assessment were recollected at the three-month follow-up (T3). We have reported all the scales and questionnaires administered at the various assessment steps (AAE, OAE, T1, and T3) in Supplementary material 2. More specifically, for each instrument, we indicated the literature reference, the linking with the International Classification of Functioning (ICF) domain and chapter (34), the assessed variable, the assessor, and the assessment step of administration (AAE, OAE, T1, and T3).

The primary endpoint was the total number of falls in each group over 12 months. A fall was defined as an “unexpected event where a person inadvertently comes to rest on the ground, floor, or lower level” (35, 36). The secondary endpoints were other fall-related indicators (fall rate of subjects with one or more falls, fall severity, fall probability, and time to the first fall) recorded at the twelve-month follow-up.

All participants were provided with their fall diary and were followed up for 12 months with monthly telephone contacts to record the primary and secondary endpoints. During these monthly calls, each participant was inquired about any incurred falls at each contact, with date, circumstances, underlying cause, and related injuries. The primary endpoint was further verified at the end of the study by returning the fall diary.

The blindness of the assessments was guaranteed with various strategies:

- For both pre-test and post-test evaluations, as the former was performed before randomization, whereas the latter was undertaken by the other center's assessors, unaware of the allocation arms of the participants' within the enrolling center;
- Furthermore, subjects in both groups were instructed not to discuss their allocation with other participants and assessors during the post-test assessments;
- Finally, the assessor was unaware of the allocation arms at the monthly follow-up calls.

### Statistical analyses

#### Sample size calculation

The sample size was determined based on the assumptions that the fall risk in the control group was equal to 50% and that the experimental intervention was able to reduce this risk by 30% (14, 37), that is, to reduce the fall risk in the treatment group to 35%. Fixing the type I error at 0.05 (95% confidence level) and the type II error at 0.20 (80% power), we calculated a sample size of at least 366 subjects (183 per group). Thus, if enrolled patients had a baseline risk higher than 0.5 and/or the intervention had an efficacy higher than 30%, a sample of 366 patients would have a test power higher than 80%.

Finally, it should be noted that the sample size calculation was made by considering the expected risk of falling in the enrolled population. However, the primary endpoint was represented by the rate of falls. Considering that the rate of falls is usually 15–20% higher than the risk of falling due to multiple fallers (1), the sample size estimate was largely conservative and would have accommodated up to 15–20% of subjects lost to follow-up.

#### Descriptive statistics for all participants

Descriptive statistics were calculated at the time of enrolment in the study. Summary statistics were means and standard deviations for quantitative variables, median and interquartile ranges for categorical variables, and absolute frequencies and percentages for nominal variables.

#### Primary and secondary endpoint calculations

The number of falls recorded monthly by telephone interview was the basic element for the primary endpoint calculation. In particular, the monthly fall number was added for all 12 months of follow-up to obtain the number of falls observed during the entire period of inclusion in the study of each participant.

The start and end date of the follow-up were needed to calculate the time to the first fall (secondary endpoint). Therefore, the date of randomization for each subject was considered the start date for the follow-up. The follow-up end date was calculated differently for participants with at least one fall and those without falls. For the former, we considered the least recent date among the dates of telephone interviews in which at least one fall was reported. For the latter, the most recent date among telephone interviews was considered. Thus, the follow-up time in months was equal to the difference in days between the start and end follow-up dates, divided by 30.4 (mean duration of a month).

#### Multivariate analyses for the final endpoint prediction on the whole sample

We performed multivariate analyses on the whole sample to statistically control for additional variables (i.e., experimental groups, risk classes, and gender), which could have influenced the predictive ability of the models for the primary and secondary endpoints for the whole sample. We used the following models and statistics:

- ‘Number of falls' (primary endpoint): negative binomial regression model, Incidence Rate Ratio (IRR) with a 95% confidence interval (95CI%) and a p-value;
- ‘Fall probability for one fall, two or more falls, and three or more falls (multiple fallers) within 12 months' (secondary endpoints): logistic regression model, Odds Ratio (OR) with a 95%CI and a p-value;
- ‘Time to the first fall' (secondary endpoint): Cox regression model, Hazard Ratio (HR) with a 95%CI and a *p*-value.

#### Analysis of the differences between groups (IG and CG)

Concerning the study's primary endpoint, the comparison of observed fall incidences in the two groups was performed using statistical regression methodologies for counting data. A model assumes a negative binomial distribution for the response variable (number of falls). The results were expressed as the Incidence Rate Ratio (IRR) with a 95% confidence interval (95 CI%) and a *p*-value, comparing the experimental and control groups.

Concerning the secondary endpoint “fall probability”, the results were expressed as Relative Risk (RR) with a 95%CI and a *p*-value, referring to the comparison between IG and CG. This fall probability was calculated for one fall, two or more falls, and three or more falls (multiple fallers) within 12 months.

A Cox regression model was employed to perform the analyses of the secondary endpoint “time to the first fall”. The results were expressed as Hazard Ratio (HR) with a 95%CI and a *p*-value, comparing the two groups. In addition, the cumulative probabilities of occurrence of at least one fall were graphically represented as Kaplan-Meier survival curves, reporting the survival point estimate from falls at three, six, and 12 months, with 95%CI.

We also explored the impact of risk classes and gender (additional variables) on comparing primary and secondary endpoints between the two groups. For doing so, we performed a multivariate analysis where we statistically controlled for the additional variables employing the same models and statistics indicated in the previous section on the multivariate analysis to predict the final endpoint on the whole sample.

#### Analysis of the differences between sub-groups (risk classes and gender)

The analysis of the differences between randomization arms (IG and CG) were also performed for each of the subgroups identified by the four etiological risk class categories considered in the study (age between 65 and 80, age over 80, elderly with Parkinson's Disease, elderly with a previous stroke), and by gender.

#### Analysis of the differences between groups (IG and CG) for T3 endpoints (post-test)

##### Rasch analysis

Preliminary to comparing the two groups on post-test with ANCOVA, we performed a Rasch analysis of the scales and questionnaires involved in the comparison. Rasch analysis was conducted because ANCOVA is a parametric statistical analysis requiring continuous variables, whereas the total scores of scales and questionnaires deliver ordinal data. Indeed, within Rasch analysis, it may be possible to transform the ordinal total score of a scale or a questionnaire into interval-level person estimates of ability, should the data fit the fundamental measurement requirements of the Rasch model (i.e., the mathematical model upon which Rasch analysis relies) (38). In particular, the Rasch analysis focused on the following indicators:

- FROP-Com (39);
- Berg Balance Scale [BBS (40, 41)];
- Performance-Oriented Mobility Assessment [POMA (41, 42)];
- Fullerton Advanced Balance Scale [FABS (41, 43)];
- Mini-BESTest (44).

The FROP-Com is a global fall risk indicator, while the other four indicators all quantify balance, although with differences related to the measurement range. Therefore, two Rasch analyses were carried out separately: the first for the FROP-Com and the second for the four balance indicators. Given their conceptual equivalence (41), the latter analysis treated items of single balance scales as testlets. Super-items or testlets are sum scores from a set of associated items. Thus, the Rasch analysis was conducted on four testlets, one for each balance scale (45). This approach was adopted to absorb the local dependence between the items of the various balance scales (41, 45–47).

##### Pre-test vs. post-test differences between groups (IG and CG)

The values of the above five indicators, calculated before and after the intervention, were compared between the two groups using parametric statistical techniques. In particular, we reported the mean values of these parameters at the pre-test and post-test levels. The post-intervention values were compared between the groups through a linear regression model that uses the treatment and the pre-intervention value as independent variables (ANCOVA model). This analysis was reported as mean differences (MD) with 95% and *p*-value confidence interval. The effect size was calculated as Cohen's *d* by comparing pre-post differences between groups.

#### Cases lost to follow-up

Whenever possible, the reasons for any cases lost to follow-up were recorded. Concerning logistic regression, we initially conducted an analysis that considered all randomized subjects without considering any follow-up loss according to the principle of the intention to treat. In case of loss to follow-up due to death or other causes, we considered the information collected up to that time. Whether a subject was lost to follow-up, independent from experiencing a fall or not (primary outcome), their data were considered for the analysis. Thus, it was possible to conduct sensitivity analyses that hypothesized various scenarios of the outcomes considered for loss to follow-up.

Regarding the analysis of survival curves, any loss to follow-up data was treated as censored since the last available information for these subjects. However, it was possible to conduct further sensitivity analyses even in this context.

#### Statistical software

Statistical analyses were performed using the Stata 14 software (StataCorp LP, College Station) and R 3.4.3 (the R Foundation for Statistical Computing, Wien) by the Medical Statistics Unit of the University of Modena e Reggio Emilia, using a 95% confidence level (p 0.05). In addition, Rasch analyses were carried out using the software RUMM 2030 (version 5.4 for Windows. RUMM Laboratory Pty Ltd, Perth, Australia: 1997-2017; www.rummlab.com).

## Results

### Descriptive statistics for all participants (*n =* 403)

**Figure 2:**
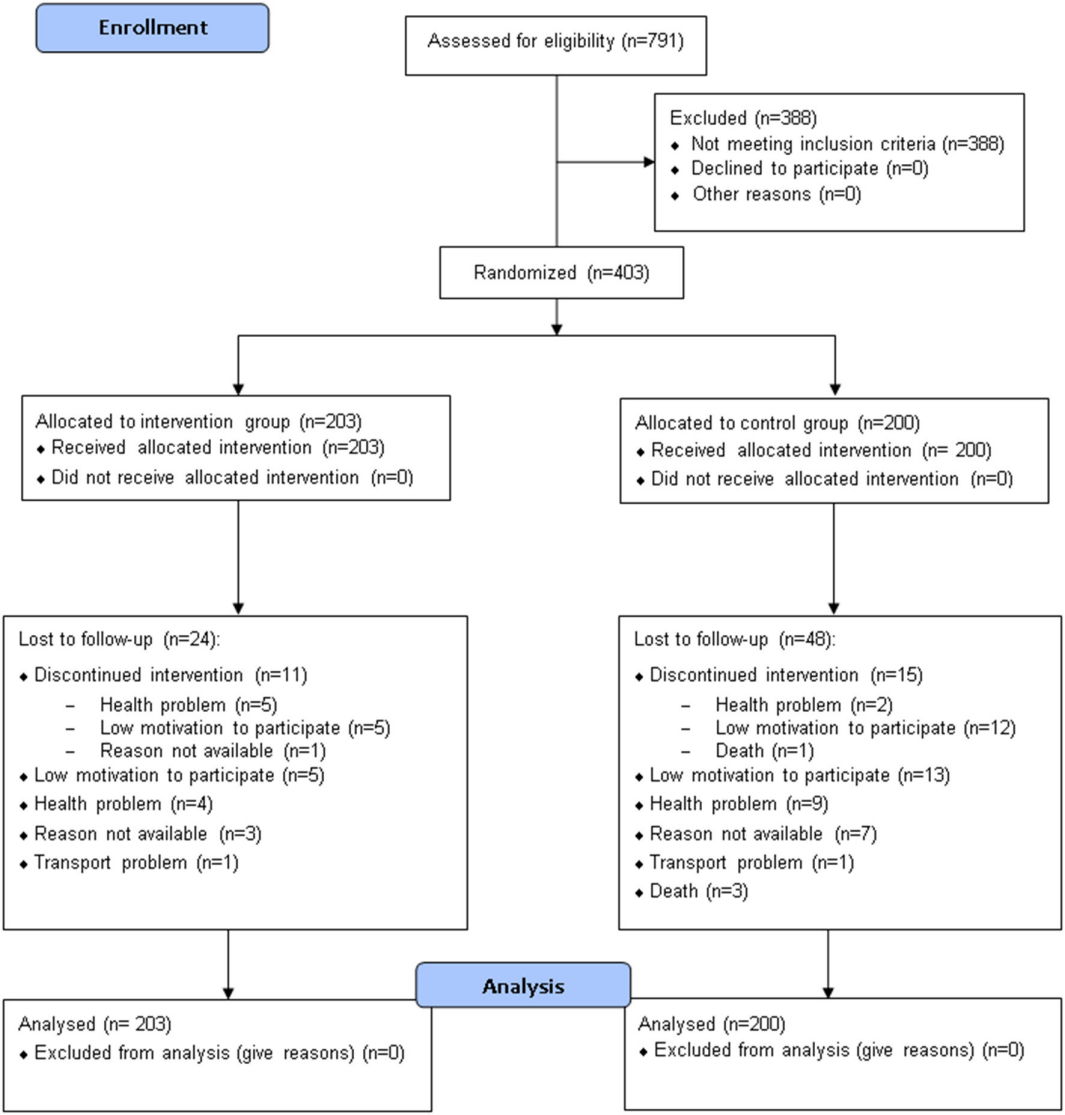
CONSORT 2010 flow diagram.

Seven hundred ninety-one participants were assessed for eligibility, and 403 were included in the study and randomized to either the CG (*n* = 200) or the IG (*n* = 203) (Table 2, Figure 2). Seventy-one subjects (48 in the CG and 23 in the IG group) were lost to follow-up (Figure 2); among them, respectively, 15 and 11 elderly people interrupted their participation during the treatment period (“discontinued intervention”, Figure 2). The two centers enrolled almost an equal number of patients (49.1% and 50.9% at Modena and Reggio Emilia, respectively).

**Table 2:** Clinical and demographic sample characteristics.

**Variable**			**Total sample**		**Control Group**		**Intervention Group**		***P*-value**
**Participants**	n	(%)	403	(100%)	200	(49.6%)	203	(50.3%)	-
**Center**									
Modena	n	(%)	198	(49.1%)	98	(49.0%)	100	(49.3%)	-
Reggio Emilia	n	(%)	205	(50.9%)	102	(51.0%)	103	(50.7%)	-
**Age (years)**	Mean	(SD)	76.2	(6.3)	76.1	(6.2)	76.3	(6.4)	n.s.
**Gender**									
Females	n	(%)	264	(65.5%)	130	(65.0%)	134	(66.0%)	n.s.
Males	n	(%)	139	(34.5%)	70	(35.0%)	69	(34.0%)	n.s.
**Education level (years)**	Mean	(SD)	9.0	(4.6)	8.7	(4.6)	9.3	(4.6)	n.s.
**Group stratification by risk classes**									
Elderly, age 65-80	n	(%)	176	(43.7%)	85	(42.5%)	91	(44.8%)	n.s.
Elderly, age >80	n	(%)	87	(21.6%)	46	(23.0%)	41	(20.2%)	n.s.
Elderly, Parkinson	n	(%)	78	(19.4%)	39	(19.5%)	39	(19.2%)	n.s.
Elderly, Stroke	n	(%)	62	(15.4%)	30	(15.0%)	32	(15.8%)	n.s.
**Level of impairment of elderly with neurological diseases**									
Modified H&Y (PD)	Median	(IQR)	3	(1)	2.5	(2)	3	(1.5)	n.s.
NIHSS (Stroke)	Median	(IQR)	2	(4)	2	(5)	2.5	(4)	n.s.
Modified LE-FMA Par (Stroke)	Median	(IQR)	31	(8)	31	(6)	30	(11)	n.s.
Modified LE-FMA No-Par (Stroke)	Median	(IQR)	34	(5)	35	(3)	34	(6)	n.s.
**Estimated fall risk (FROP-Com criterion)**									
Low	n	(%)	32	(7.9%)	15	(7.5%)	17	(8.4%)	n.s.
Medium	n	(%)	146	(36.2%)	72	(36.0%)	74	(36.5%)	n.s.
High	n	(%)	225	(55.8%)	113	(56.5%)	112	(55.2%)	n.s.

sd, standard deviation; n.s., not significant at 0.05 level; H&Y, Modified Hoehn and Yahr scale; Modified LE-FMA Par, ‘Part A: Ability to perform active movements – Lower Extremity' of the modified version of the Fugl-Meyer Motor Assessment (paretic side); Modified LE-FMA No-Par, ‘Part A: Ability to perform active movements – Lower Extremity' of the modified version of the Fugl-Meyer Motor Assessment (non-paretic side); NIHSS, National Institutes of Health Stroke Scale.

The mean age of enrolled participants was 76.2 years (SD: 6.3), and about two-thirds (65.5%) were females. The mean level of education was 9.0 years of schooling (SD: 4.6), corresponding approximately to the completion of lower secondary school. About two-thirds of the enrolled patients (65.2%) were elderly with an estimated fall risk at 1 year comprised between 33 and 50%, as 43.7 and 21.6% were classified within the 65 to 80 and >80 risk classes, respectively. The remaining 34.7% were elderly patients with an associated neurological condition. Their estimated fall risk at 1 year was between 60 and 70%, as 19.4 and 15.4% had a diagnosis of Parkinson's Disease or stroke, respectively.

Regarding the level of impairment of older adults with an associated neurological disease, those with PD showed a median value of the modified Hoehn and Yahr scale (H&Y) of 3 (Interquartile range IQR: 1), thus suggesting that almost 50% of them were physically independent. Subjects with stroke had a median value of the National Institutes of Health Stroke Scale (NIHSS) of 2 (IQR: 4), highlighting that almost 50% of these elderly suffered from a minor stroke's sequelae (Table 2). In particular, persons with stroke had a median value of the ‘Part A: Ability to perform active movements – Lower Extremity' of the modified version of the Fugl-Meyer Motor Assessment (modified LE-FMA, range 0–36 each side) of 31 for the paretic lower limb and 34 for the non-paretic one. These data suggest that almost 50% of them had a motor impairment in active movements on the paretic side and that a minimum of these persons also had a mild motor impairment on the non-paretic side.

Considering the estimated fall risk of the enrollment patients, only 7.9% could be considered at ‘low risk' according to the FROP-Com. In contrast, the enrollment algorithm classified the remaining 92.1% of patients correctly as being at moderate (36.2%) or high fall risk (55.8%) according to the FROP-Com. All subjects' characteristics at baseline for each group (IG and CG) were reported in Table 2. Besides, those characteristics were balanced between the two groups (Table 2), with no statistically significant difference detected.

Amongst the participants, the majority (58.6%) experienced at least one or more falls. In particular, 21.8% and 14.6% experienced one or two falls, respectively, whereas the percentage of multiple fallers (>2 falls) was 22.1% (Table 3). The rate of fallers, defined as those with at least two falls, was about one-third (36.7%). Regarding the primary endpoint, six hundred ninety falls were reported at the twelve-month follow-up (Table 3), with a mean number of falls per participant equal to 1.71 (SD 3.36).

**Table 3:** Endpoint evaluation between groups (IG and CG).

			**Total**		**Control group**		**Intervention group**		***P*-value**
**Participants**	n	(%)	403	-	200		203		-
**Participants by falls (no falls vs**. **≥1 falls)**									
0 falls	n	(%)	167	(41.4%)	83	(41.5%)	84	(41.4%)	n.s.
≥1 falls	n	(%)	236	(58.6%)	117	(58.5%)	119	(58.6%)	n.s.
One fall	n	(%)	88	(21.8%)	40	(20.0%)	48	(23.6%)	n.s.
Two falls	n	(%)	59	(14.6%)	26	(13.0%)	33	(16.3%)	n.s.
More than two falls (multiple fallers)	n	(%)	89	(22.1%)	51	(25.5%)	38	(18.7%)	n.s.
**Participants by falls (0–1 falls vs**. **≥2 falls)**									
No fallers (0-1 falls)	n	(%)	255	(63.3%)	123	(61.5%)	132	(65.0%)	n.s.
Fallers (≥2 falls)	n	(%)	148	(36.7%)	77	(38.5%)	71	(35.0%)	n.s.
**Fall rate (primary endpoint)**									
Total number of falls	n	(%)	690	-	353	(51.2%)	337	(48.8%)	n.s.
Mean number of falls per participant	Mean	(SD)	1.71	(3.33)	1.77	(3.17)	1.66	(3.49)	n.s.
**Falls by injury**									
No injury	n	(%)	465	(67.4%)	238	(67.4%)	227	(67.4%)	n.s.
Minor injury, no medical consultation	n	(%)	106	(15.4%)	57	(16.2%)	49	(14.5%)	n.s.
Minor injury, with medical consultation	n	(%)	75	(10.9%)	34	(9.6%)	41	(12.2%)	n.s.
Serious injury	n	(%)	44	(6.4%)	24	(6.8%)	20	(5.9%)	n.s.
**Probability of absence of falls (Kaplan-Meier)**									
Time to the first fall in months	Median	(95%CI)	11.1	(9.4–12.3)	11.1	(7.6–12.3)	11.2	(9.7–NA)	n.s.
at 3 months	%	(95%CI)	77.6%	(73.7–81.8%)	76.5%	(70.8–82.6%)	78.8%	(73.4–84.6%)	n.s.
at 6 months	%	(95%CI)	65.0%	(60.5–69.8%)	63.4%	(57.0–70.4%)	66.5%	(60.3–73.3%)	n.s.
at 12 months	%	(95%CI)	47.3%	(42.6–52.4%)	46.8%	(40.3–54.2%)	47.8%	(41.4–55.2%)	n.s.

sd, standard deviation; CI95%, confidence interval at 95% level; NA, not applicable; n.s., not significant at 0.05 level.

Most falls (67.4%) led to no injury, whereas 32.6% were associated with various injuries. In particular, 6.4% of them led to serious injury requiring hospitalization, whereas 10.9 and 15.4% of falls were associated with minor injury either requiring or not a medical consultation, respectively.

The median time of occurrence of the first fall was 11.1 months. A probability of absence of falls of 77.6% (95%CI [73.7, 81.8%]), 65.0% (95%CI [60.5, 69.8%]) and 47.3% (95%CI [42.6, 52.4%]) were recorded, respectively, at three, six, and twelve months. All these endpoints (reported in Table 3) did not show any statistically significant difference between the two groups.

### Multivariate analyses for the final endpoint prediction on the whole sample

Regarding the “risk class” effect on the whole sample, after statistically controlling for the additional variables “experimental group” and “gender”, the elderly with PD showed a significantly higher risk for all the considered endpoints compared to the reference category “age 65-80” (Table 4). Moreover, the inclusion in the PD risk class was the strongest predictor for all the endpoints. In particular, the number of falls for these subjects was 2.18 higher than those aged 65**–**80 after controlling for the cited variables.

**Table 4:** Multivariate analyses for the final endpoint prediction on the whole sample.

**Endpoint**	**Statistics**	**Estimate**	**95 CI%**		***p*-value**
**Number of falls**					
Experimental groups					
Control	-	-	-	-	-
Treatment	IRR	0.94	0.71	1.25	0.676
Risk classes					
Age 65–80	-	-	-	-	-
Age >80	IRR	1.10	0.75	1.60	0.639
Parkinson	IRR	2.18	1.47	3.23	0.000
Stroke	IRR	0.66	0.41	1.04	0.076
Gender					
Female	-		-	-	-
Male	IRR	1.74	1.27	2.40	0.001
**Fall probability (1 fall)**					
Experimental groups					
Control	-		-	-	-
Treatment	OR	0.88	0.59	1.32	0.536
Risk classes					
Age 65–80	-		-	-	-
Age >80	OR	1.30	0.77	2.19	0.330
Parkinson	OR	2.35	1.28	4.35	0.006
Stroke	OR	0.54	0.29	0.99	0.047
Gender					
Female	-		-	-	-
Male	OR	0.87	0.55	1.39	0.570
**Multi fall probability (≥2 falls)**					
Experimental groups					
Control	-		-	-	-
Treatment	OR	0.84	0.55	1.29	0.437
Risk classes					
Age 65–80	-		-	-	-
Age >80	OR	1.72	0.99	3.00	0.054
Parkinson	OR	3.46	1.90	6.29	0.000
Stroke	OR	0.99	0.50	1.97	0.983
Gender					
Female	-		-	-	-
Male	OR	1.16	0.72	1.87	0.549
**Multi fall probability (≥3 falls)**					
Experimental groups					
Control	-		-	-	-
Treatment	OR	0.59	0.35	1.00	0.052
Risk classes					
Age 65–80	-		-	-	-
Age >80	OR	1.79	0.91	3.53	0.091
Parkinson	OR	4.14	2.11	8.11	0.000
Stroke	OR	0.48	0.17	1.35	0.162
Gender					
Female	-		-	-	-
Male	OR	1.73	0.98	3.04	0.058
**Time to the first fall**					
Experimental groups					
Control	-	-	-	-	-
Treatment	HR	0.88	0.68	1.15	0.370
Risk classes					
Age 65–80	-	-	-	-	-
Age >80	HR	0.88	0.84	1.66	0.326
Parkinson	HR	1.18	1.21	2.48	0.003
Stroke	HR	0.65	0.41	1.04	0.072
Gender					
Female	-	-	-	-	-
Male	HR	0.93	0.68	1.26	0.619

CI95%, Confidence Interval at 95% level; IRR, Incidence Rate Ratio; OR, Odds Ratio; HR, Hazard Ratio.

Each line constitutes an independent variable in the multivariate model for predicting each specific endpoint on the whole sample. The first line of each considered macro variable group (i.e., Control for Experimental groups, Age 65-80 for Risk classes, and Female for Gender) represents the reference category with which the comparison was made.

Furthermore, regarding “fall probability”, they were 2.35 times more likely to have at least one fall, 3.46 to have at least two falls, and over four times to have three or more falls compared to the reference category. Finally, subjects with PD had an HR of 1.18 to fall first compared to the 65–80 risk class. Besides, concerning ‘fall probability' for two or more falls, the elderly aged over 80 demonstrated a higher risk of falling (borderline significance) in comparison to younger subjects aged 65-80 (OR = 1.72, 95%CI [0.99–3.00], *p* = 0.054). On the other hand, people with stroke seemed to have a lower risk of falling one or more times (borderline significance) in comparison to those aged 65–80 (OR=0.54, 95%CI [0.29–0.99], *p* = 0.047) (Table 4).

Concerning the “gender” effect on the whole sample, after statistically controlling for the additional variables “experimental groups” and “risk classes”, males reported a significantly higher risk for “the number of falls” and the “fall probability” for three or more falls than females. They had a “number of falls” 1.74 higher, and they were 1.73 times more likely (borderline significance) to have three or more falls than females (Table 4).

### Differences between groups (CG and IG)

**Figure 3:**
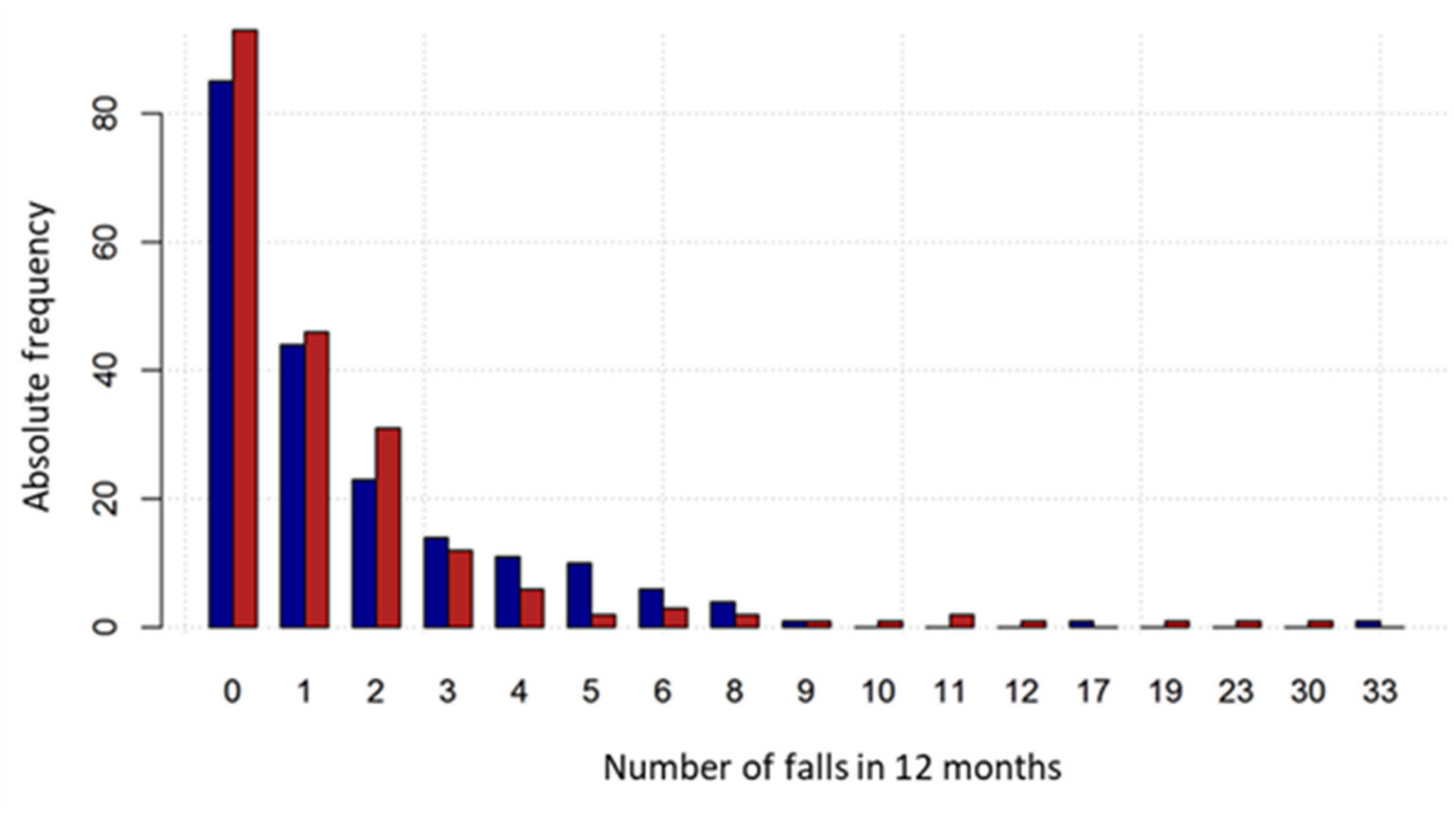
Fall number distribution by group. The intervention group is represented by the red bars and the control group by the blue bars.

In the CG and the IG, most participants (58.5 and 58.6%, respectively) fell at least once or more. In particular, 20.0% and 23.6% experienced one fall, 13.0 and 16.3% two falls, whereas the percentage of multiple fallers (>2 falls) was 25.5 and 18.7%, respectively (Table 3). The percentage of fallers with at least two falls was 38.5% and 35% in the CG and the IG, respectively. Regarding the primary endpoint, 353 falls were reported at the twelve-month follow-up in CG, compared to 337 in the IG (Table 3), with a mean number of falls per participant equal to 1.77 (SD 3.17) and 1.66 (SD 3.49) falls, respectively. Fall distribution by groups is reported in Figure 3.

Most falls (67.4%) led to no injury for both groups, whereas the remaining one-third was associated with various degrees of injury. In particular, 16.2% in the CG and 14.5% in the IG of falls led to minor injuries not requiring medical consultation, whereas 9.6% and 12.2% of falls were associated with minor injuries requiring medical consultation. Finally, 6.8% of falls in the CG and 5.9% in the IG led to serious injury requiring hospitalization (Table 5).

**Table 5:** Analysis of observed differences between IG and CG.

**Endpoint**	**Statistic**	**Estimate**	**CI 95%**		***p*-value**
Number of falls	IRR	0.94	0.69	1.29	0.693
Fall probability (1 fall)	RR	0.94	0.79	1.12	0.503
Multi fall probability (≥2 falls)	RR	0.89	0.67	1.17	0.398
Multi fall probability (≥3 falls)	RR	0.68	0.45	1.01	0.052
Time to the first fall	HR	0.89	0.69	1.16	0.398

CI95%, Confidence Interval at 95% level; IRR, Incidence Rate Ratio; RR, Relative Risk; HR, Hazard Ratio.

The association measures are expressed as a comparison between the intervention and control groups.

A probability of absence of falls of 76.5% (95%CI [70.8, 82.6%]) and 78.8% (95%CI [73.4, 84.6%]) in the first 3 months were recorded, respectively, for CG and IG. No statistically significant differences were observed between groups regarding the number of falls (Incidence Rate Ratio–IRR = 0.94, 95%CI [0.69-1.29], *p* = 0.693), and the fall probability for one fall, and two or more falls (Risk Ratio one fall - RR=0.94, 95%CI [0.79–1.12], *p* = 0.503; Risk Ratio two or more falls–RR=0.89, 95%CI [0.67–1.17], *p* = 0.398). Regarding the falling probability for three or more falls (multiple fallers), this was in favor of the IG, although slightly above the chosen level of statistical significance (Risk Ratio three or more falls–RR=0.68, 95%CI [0.45–1.01], *p* = 0.052) (Table 5).

The median time to the first fall was 11.1 months (95%CI 7.6–12.3) in the CG and 11.2 months (95%CI 9.7–NA) in the CG. No statistically significant differences were observed between groups regarding the time to the first fall (Hazard Ratio – HR = 0.89, 95%CI [0.69–1.16], *p* = 0.398) (Table 4).

The multivariate analysis, after statistically controlling for the additional variables “risk classes” and “gender”, found no statistically significant differences in favor of IG regarding the number of falls (IG Incidence Rate Ratio – IRR=0.94, 95%CI [0.71–1.25], *p* = 0.676), and the “fall probability” for one fall, and two or more falls (IG Odds Ratio one fall – OR=0.88, 95%CI [0.59 – 1.32], *p* = 0.536; IG Odds Ratio two or more falls – OR=0.84, 95%CI [0.55–1.29], *p* = 0.437). However, regarding the falling probability for three or more falls (multiple fallers), the probability was in favor of the IG even in this analysis, although slightly above the chosen level of statistical significance (IG Odds Ratio for three or more falls – OR = 0.59, 95%CI [0.35–1.00], *p* = 0.052) (Table 4). Besides, no statistically significant differences were observed in favor of IG regarding the time to the first fall (IG Hazard Ratio - HR=0.88, 95%CI [0.68-1.15], *p* = 0.370) (Table 4).

### Differences between sub-groups (risk classes and gender) in the CG and IG

Regarding the number of falls (Table 6), the comparison between CG and IG showed a trend toward a lower (although not significant) risk for elderly aged 65–80 (IRR=0.79, 95%CI [0.50, 1.25]), elderly aged >80 (IRR = 0.85, 95%CI [0.51, 1.40]), and elderly with associated Parkinson's Disease (IRR=0.94, 95%CI [0.52, 1.72]) in the IG. In addition, the risk appeared lower (although not significant) for the elderly with stroke sequelae (IRR = 2.39, 95%CI [0.88, 6.49]) in the CG and for the “female” gender subgroup (IRR = 0.73, 95%CI [0.53, 1.02]; borderline significance) within the IG.

**Table 6:** Analysis of observed differences between subgroups in the IG and CG.

**Endpoint**	**Statistics**	**Estimate**	**95 CI%**		***p*-value**
**Number of falls**					
Risk classes					
Age 65–80	IRR	0.79	0.50	1.25	0.313
Age >80	IRR	0.85	0.51	1.40	0.519
Parkinson	IRR	0.94	0.52	1.72	0.849
Stroke	IRR	2.39	0.88	6.49	0.086
Gender					
Female	IRR	0.73	0.53	1.02	0.063
Male	IRR	1.20	0.68	2.13	0.530
**Fall probability (1 fall)**					
Risk classes					
Age 65–80	RR	0.86	0.65	1.13	0.276
Age >80	RR	1.03	0.74	1.47	0.829
Parkinson	RR	0.93	0.70	1.23	0.615
Stroke	RR	1.22	0.63	2.35	0.553
Gender					
Female	RR	0.89	0.72	1.11	0.307
Male	RR	1.04	0.77	1.40	0.791
**Multi fall probability (≥2 falls)**					
Risk classes					
Age 65–80	RR	0.72	0.33	1.55	0.397
Age >80	RR	0.52	0.22	1.24	0.125
Parkinson	RR	0.62	0.36	1.05	0.068
Stroke	RR	3.75	0.44	31.7	0.185
Gender					
Female	RR	0.83	0.58	1.21	0.334
Male	RR	0.98	0.65	1.48	0.917
**Multi fall probability (≥3 falls)**					
Risk classes					
Age 65–80	RR	0.85	0.51	1.42	0.542
Age >80	RR	1.21	0.65	1.95	0.682
Parkinson	RR	0.72	0.48	1.19	0.111
Stroke	RR	1.21	0.51	2.83	0.667
Gender					
Female	RR	0.56	0.31	1.00	0.048
Male	RR	0.83	0.49	1.41	0.487
**Time to the first fall**					
Risk classes					
Age 65–80	HR	0.83	0.56	1.25	0.381
Age >80	HR	0.92	0.54	1.59	0.776
Parkinson	HR	0.83	0.49	1.41	0.497
Stroke	HR	1.32	0.58	3.01	0.509
Gender					
Female	HR	0.82	0.59	1.13	0.216
Male	HR	1.03	0.66	0.61	0.895

CI95%, Confidence Interval at 95% level; IRR, Incidence Rate Ratio; RR, Relative Risk; HR, Hazard Ratio.

The association measures are expressed as a comparison between the subgroups in the IG and CG.

Concerning the falling probability for one fall, elderly aged 65–80 and with PD in the IG had a lower (although not significant) probability of falling than those randomized in the CG (RR=0.86, 95%CI [0.65, 1.13] and, respectively, RR=0.93, 95%CI [0.70, 1.23]). On the other hand, elderly aged >80 and elderly with stroke had a higher (although not significant) probability of falling if randomized in the IG (RR=1.03, 95%CI [0.74, 1.47] and, respectively, RR=1.22, 95%CI [0.63, 2.35]). Furthermore, the falling probability appeared lower (although not significant) for the ‘female' gender subgroup within the IG (IRR=0.89, 95%CI [0.72, 1.11]), as shown in Table 6.

Concerning the falling probability for two or more falls, elderly aged 65–80 and aged >80 in the IG had a lower (although not significant) probability of falling than those randomized in the CG (RR = 0.72, 95%CI [0.33, 1.55] and, respectively, RR = 0.52, 95%CI [0.22, 1.24]). Even elderly with PD in the IG had a lower (although not significant) probability of falling than those randomized in the CG, with a RR closer to the chosen level of statistical significance (RR = 0.62, 95%CI [0.36, 1.05]). Differently, the elderly with stroke had a higher (although not significant) probability of falling if randomized in the IG (RR = 3.75, 95%CI [0.44, 31.7]). The multi-falling probability for two or more falls appeared lower (although not significant) again for the ‘female' gender subgroup within the IG (IRR = 0.83, 95%CI [0.58, 1.21]) (Table 6).

Regarding the falling probability for three or more falls (multiple fallers) (Table 6), similarly to the probability for one fall, elderly aged 65–80 and with PD in the IG had a lower (although not significant) probability of falling than those randomized in the CG (RR = 0.85, 95%CI [0.51, 1.42] and, respectively, RR = 0.72, 95%CI [0.48, 1.19]). On the other hand, elderly aged >80 and with stroke had a higher (although not significant) probability of falling if randomized in the IG (RR = 1.21, 95%CI [0.65, 1.95] and, respectively, RR = 1.21, 95%CI [0.51, 2.83]). As for the previous falling probabilities, the falling probability for three or more falls also appeared significantly lower for the “female” gender subgroup within the IG (IRR = 0.56, 95%CI [0.31, 1.00]).

Considering the endpoint ‘time to the first fall' (Table 6), elderly aged 65–80, elderly aged >80, and elderly with Parkinson's had a lower (although not significant) hazard ratio if randomized in the IG in comparison to the CG (HR = 0.83, 95%CI [0.56, 1.25]); HR = 0.92, 95%CI [0.54, 1.59]; HR = 0.83, 95%CI [0.49, 1.41], respectively), Instead, the hazard ratio was higher (HR = 1.32, 95%CI [0.58, 3.01]) for elderly with stroke sequelae randomized in the IG. Finally, as expected, the HR appeared lower (although not significant) for the “female” gender subgroup within the IG (IRR = 0.82, 95%CI [0.59, 1.13]).

### Rasch analysis

The final solutions for the FROP-Com and the balance scales showed adequate fitness to the Rasch Model (Table 7). Hence, it was possible to devise conversion tables from ordinal scores to interval-level measurements (having, as a unit of measurement, the logit), then used for the subsequent analysis.

**Table 7:** Rasch analysis results (final analyses) on scales used for pre-test vs. post-test differences analysis.

**Analysis**	**Description of analysis**	**Fitness to the rasch model**						**Targeting**		**Separation reliability**	
		**Fit residual items**		**Fit residual persons**		**Item-trait interaction**		**Person location**			
		**Mean**	**SD**	**Mean**	**SD**	** $\chi_{\text{df}}^{2}$ **	**P**	**Mean**	**SD**	**PSI**	**α**
FROP-Com	Final analysis	−0.322	1.557	−0.483	0.810	26.160_36_	0.509	−0.660	0.555	0,711	-
Balance scales	Final analysis	1.260	4.697	0.418	0.991	42.647_27_	1.202	1.045	1.169	0,962	-
*Expected values*		*0*	*1*	*0*	*1*		*n.s*.	*≈0*	*-*	*≥0.900: person measurement*	
										*≥0.700: group measurement*	

FROP-Com, Fall Risk for Older People living in the Community; SD, standard deviation; df, degrees of freedom; P, Bonferroni-corrected p-value; PSI, person separation index; α, Cronbach's alpha.

### Pre-test vs. post-test differences between groups (CG and IG)

The ANCOVA analysis (Table 8) showed no significant difference between the CG and the IG for the post-test FROP-Com (MD = −0.03 logits, 95% CI [−0.13, 0.07]), BBS (+0.15 logits, 95% CI [−0.13, 0.07]), and POMA measures (+0.12 logits, 95%CI [−0.14, 0.37]) after controlling for the pre-test values.

**Table 8:** Analysis of pre-test vs. post-test differences for FROP-Com and balance indicators.

**Variable**		**Control group**			**Intervention group**			**Comparison**		**Effect size**
		**mean**	**sd**	**n**	**mean**	**sd**	**n**	**MD (CI95%)**	**p**	**Cohen's d (CI 95%)**
FROP-Com	Pre	−0.5	0.5	200	−0.5	0.5	203			
	Post	−0.9	0.6	132	−0.9	0.6	153			
	Post–pre	−0.3	0.4	132	−0.3	0.4	153	−0.03 (−0.13; 0.07)	0.543	−0.08 (−0.32; 0.15)
BBS	Pre	2.2	1.6	200	2.1	1.6	203			
	Post	2.5	1.8	156	2.6	1.8	184			
	Post –pre	0.1	2.7	156	0.5	2.3	184	0.15 (−0.23; 0.53)	0.445	0.14 (−0.08; 0.35)
POMA	Pre	2.6	1.8	200	2.6	1.9	203			
	Post	3.0	1.9	156	3.1	1.9	182			
	Post–pre	0.2	1.2	156	0.3	1.3	182	0.12 (−0.14; 0.37)	0.363	0.10 (−0.11; 0.31)
MBT	Pre	0.6	2.3	198	0.4	2.4	200			
	Post	1.0	2.5	156	1.2	2.7	182			
	Post-pre	0.2	1.8	155	0.6	2.0	179	**0.42 (0.03; 0.81)**	**0.035**	**0.26 (0.04; 0.48)**
FABS	Pre	0.4	1.2	200	0.4	1.2	203			
	Post	0.4	1.2	156	0.4	1.3	184			
	Post-pre	0.1	0.7	156	0.3	0.7	184	**0.21 (0.06; 0.36)**	**0.006**	**0.32 (0.10; 0.53)**

sd, standard deviation; MD, mean differences in post-test values adjusted for pre-test values; CI95%, Confidence Interval at 95% level; FROP-Com, Fall Risk for Older People in the Community; BBS, Berg Balance Scale; POMA, Performance Oriented Mobility Assessment; FABS, Fullerton Advanced Balance Scale; MBT, Mini-BESTest.

In bold, significant pre-test vs. post-test differences were reported.

However, there were statistically significant differences for the post-test MBT measures (MD = +0.42 logits, 95%CI [0.03, 0.81], *p* = 0.035; effect size 0.26, 95%CI [0.04; 0.48]) and FABS (MD = +0.21 logits, 95%CI [0.06, 0.36], *p* = 0.006; effect size 0.32, 95%CI [0.10; 0.53]) between the two groups after controlling for the pre-test measures.

## Discussion

In the PRE.C.I.S.A. RCT study, we evaluated the efficacy of the simultaneous administration of a multiple-component and a multifactorial personalized intervention in reducing fall rates in community-dwelling older adults at moderate-to-high fall risk compared to usual care. Another peculiar aspect of the study was the inclusion in the sample of elderly with an even higher fall risk because of a concurrent diagnosis of Parkinson's Disease or stroke sequelae. The results showed no statistically significant differences between groups regarding the number of falls, the falling probability, and the time to the first fall at a twelve-month follow-up. There were no significant differences also according to the subgroup analyses. However, a lower number of falls, lower fall rates in multiple fallers, a lower mean number of falls per participant, and a lower rate of fall-related severe injuries were recorded for the intervention group, although the differences were not significant. In addition, the inclusion in the Parkinson's Disease risk class was the strongest significant predictor for all the considered endpoints in the whole sample. Finally, a significant improvement for two balance-related indicators at post-test was recorded in the intervention after controlling for the pre-test measures.

Several previous studies investigated the effects of different interventions on fall prevention in community-dwelling older adults. However, their effectiveness in reducing falls and their highly disabling consequences is still controversial (35, 48, 49). Moreover, no evidence was available on the simultaneous administration of multiple-component and multifactorial interventions to manage fall risk (14). Furthermore, RCTs aiming at reducing falls in the elderly usually exclude those with an even higher risk of falling because of an associated neurological condition, such as Parkinson's Disease (PD) and stroke. Thus, this trial was built upon two untested yet innovative hypotheses. First, as in the Prevquedas Brazil Trial (14), we hypothesized combining a multiple-component intervention with a personalized multifactorial intervention could reduce fall rates in community-dwelling older adults. Secondly, considering that most of the risk factors for falling are independent of the diseases associated with falls, we hypothesized that most of those risk factors could be targeted by the same interventions independently from the participant's risk class. Considering that the devised multicomponent intervention also included elements of physical exercise that were disease-specific, we were able to enroll the elderly with neurological conditions who were at a high risk of falling, such as those affected by PD and stroke sequelae (50–58).

According to our results, the total number of recorded falls was substantial (690), with a fall incidence of 58.6% and a mean of 1.7 (SD: 3.4) falls accounted for each included subject. These data are in contrast with those reported in the literature, where the overall fall incidence in older adults over 65 years is around 28 to 35% and about 32 to 42% in those over 75 years (59), with 0.2–1.6 falls for each included subject (2). Furthermore, the prevalence of ‘multiple fallers' observed in our trial (22.1%) was higher than that reported by previous studies (15%) in older adults (55). Indeed, several subjects reported more than 10 falls in our sample while participating in the trial, with an individual subject reporting up to 33 falls (2.8 falls per month). The observed discrepancies between our results and the literature data may be explained considering the inclusion of participants affected by neurological conditions, which are well known to be associated with multiple falls. In particular, according to previous studies, the fall risk is around 50% in Parkinson's Disease (60) and 43–70% in stroke (61). Indeed, the literature's reported incidence of multiple fallers among persons with neurological conditions is around 15% in stroke subjects (61) and over 50% in PD, where up to 13% of patients fall more than once a week (55). Besides, in our trial, the inclusion in the Parkinson's Disease risk class was the strongest significant predictor for all the considered endpoints in the whole sample, showing a risk at least 1.5 times higher for all endpoints compared to people 65–80 years old. On the other hand, the prevalence of severe injuries in our sample (6.8% in the CG and 5.9% in the IG; 6.4% for the whole sample) was instead similar to the value (10%) reported for the elderly population (59).

The statistical analyses revealed no significant differences in fall rates and related parameters between IG and CG, i.e., fall severity, fall probability for one, two or more, and three or more falls within 12 months, and time to the first fall. These results align with Lamb's and Cattaneo's works (15, 48). However, a systematic (although not significant) trend of better outcomes was reported for the IG compared to the CG. In particular, we recorded fewer falls, lower fall rates in multiple fallers, a lower mean number of falls per participant, and a lower rate of fall-related severe injuries in the IG. Furthermore, considering the falling probability for three or more falls (multiple fallers), it was in favor of the IG group slightly above the chosen level of statistical significance. These results were also confirmed with the multivariate analysis on the whole sample, where we statistically controlled for additional variables, such as risk classes and gender.

The subgroup analyses yielded similar results, with the absence of significant differences between the CG and IG regarding the number of falls, the falling probability, or the time to the first fall (all p > 0.05) across all the considered subgroups (risk classes: older adults 65–79 years, older adults ≥80 years, older adults with PD, older adults with stroke; gender: female, male). As per the general group, there was a general trend of better outcomes for the IG group, although not significant. In particular, it is interesting that the falling probability for two or more falls in the elderly with PD was very close to the level of statistical significance, considering that this was the subgroup showing a significantly higher risk for all the considered endpoints in the multivariate analysis. The stroke subgroup made an exception, as the risk of falling was apparently higher (although not significant) for patients enrolled in the IG. The fall risk for persons with stroke is notoriously high, being reported to be around 43 to 70% in the previous trials (61). Undoubtedly, intravenous thrombolysis (62) and endovascular treatments for large vessel occlusions (63) have significantly improved ischemic stroke's survival and long-term functional outcome. However, their impact on balance and other fall risk factors is unclear. Our results could also be explained in light of the literature data, which report an increase in the exposure to circumstances leading to falls (and, thus, an increase in the number of falls) brought about by an increase in physical activity (64).

Literature data about gender influence on the risk of falling suggests that females may be at a higher risk of falling than men. In particular, Stevens and Sogolow (65) showed that U.S. women older than 65 were disproportionately at higher risk than U.S. men for non-fatal fall-related injuries. These data were confirmed by Pereira et al. (66), who found that women were more susceptible to falling than men, presumably due to their poorer health and physical fitness. However, after adjusting for comorbidities, balance, lean and fat mass, they also reported that men older than 50 had a higher risk of falling than women (66). Furthermore, Johansson et al. (67) highlighted a higher fall risk for women older than 70 years compared to their male counterparts, associated with the increased variation in gait pattern during dual-task activities, which may contribute to women's greater fracture risk.

Regarding gender influence, our data showed a general trend for better outcomes within the IG group for females, although non-statistically significant. In particular, the falling probability for three or more falls in females was slightly below the statistical significance. Furthermore, the possible protective effect of the female gender emerged from the multivariate analysis performed on the whole sample for three of the considered endpoints (number of falls, fall probability for two or more falls, and three or more falls) after controlling for experimental groups and risk class. Thus, the intervention may have further reduced female subjects' risk-taking behaviors and occupational hazards, which are known to be less prevalent in women than men (36). However, some sample-specific characteristics, such as that two-thirds of the participants were females, may have played a role in producing these results. Thus, our results should be compared cautiously with literature data.

The analysis of the differences between baseline (T1) and 3-month follow-up (T3) across CG and IG was preceded by a Rasch Analysis. The latter was performed because scales' total scores are ordinal and, as such, should not be used with parametric statistical techniques such as ANCOVA (68, 69), as this may lead to erroneous results (70). The Rasch analysis allowed to elaborate conversion tables of the scales' total scores into invariant interval-level estimates of ability (whose unit of measurement is the logit) that satisfy the mathematical requirements of a general measurement theory called Additive Conjoint Measurement (71, 72). In other words, interval-level estimates produced by Rasch analysis are comparable in measurement properties to those delivered by instruments measuring physical variables, such as a thermometer. Thus, those interval-level estimates were employed for the subsequent analysis of covariance. After adjusting for the baseline values, the post-test measurements of a comprehensive fall risk indicator (FROP-Com) and four balance scales were compared between the CG and IG. In this way, we could compare the differences between groups ascribable to the administered intervention without introducing biases due to using ordinal metrics with a parametric statistical method such as ANCOVA, which requires continuous measures.

In a previous study evaluating the effects of a home-based exercise program in reducing falls in the elderly population, Vogler et al. observed an improvement in reducing fall risk and balance indicators at the end of the 12-week treatment and a subsequent return to baseline values after 24 weeks (73). Indeed, the ANCOVA showed a significant effect on balance within the intervention group only for two of the four balance indicators (FABS and Mini-BESTest). This result could be explained considering that the latter indicators are more challenging in balance ability than BBS and POMA (41, 74). In other words, no effect was likely shown with BBS and POMA because the ability range of the sample was higher than the difficulty level of the two scales. Despite the short-term effectiveness of the intervention, the small effect size of the balance change suggests that the ratio between treatment benefits and costs of administering physical exercise for the overall study duration (12 months), may be unfavorable, as indicated by some authors (73).

Indeed, the main hypothesis behind this study was that all the proposed interventions could contribute equally to avoiding the detraining effect by facilitating the adoption of a habit of regularly performing exercise and physical activity. However, the study results seem to contradict this hypothesis. First, there was no significant reduction of the overall burden of fall risk factors on post-test, as shown by the results of ANCOVA performed on FROP-Com. Second, participants in the IG provided informal positive feedback on some but not all the interventions. Indeed, the activities involving social participation, such as group exercise, educational sessions, and physiotherapy home visits, were particularly appreciated. This appreciation could be explained considering that these activities also offered socializing opportunities, thus contrasting the social isolation, which, *per se*, might be a fall risk factor (75) and may have a significant negative impact on the health and well-being of older people (76). At the same time, this could indirectly indicate a lower appreciation of the home physical exercise program. Finally, if the home exercise program was not integrated early into the participants' daily routine, this could have led, in turn, to a lower engagement at home. The latter may have facilitated a detraining effect after the eleven treatment weeks, thus losing any eventual long-term beneficial impact of the combined treatment strategy.

### Study limitations

The present study results should be considered in light of some limitations. First, we did not plan to collect any specific data on adherence to the proposed interventions, although it is reported in the literature that ‘full adherence to a fall prevention program brought significant benefits to participants, such as fewer falls and less utilization of health care resources' (77). On the other hand, the elderly at high risk of falling tend to have high attendance rates in these programs due to their higher intrinsic motivation to prevent catastrophic accidents (78). In this trial, an indirect source of information about adherence to the intervention was constituted by the physical activity report diaries suggesting lower adherence rates to physical exercise in the home setting for the Treatment Group. However, it cannot be excluded that the information provided by participants was not reliable enough. For this reason, the use of wearable sensors (79), i.e., portable inertial measurement units, to continuously record the subject's activity and adherence to home physical exercise, together with remote telemedicine support, could be strategic to achieving participants' compliance to treatment and better monitor executed exercises. Further studies should be conducted to explore fall risk management properly in a similar scenario.

Second, the home fall diaries reporting was not accurate enough. Participants were expected to report each occurred fall accurately in the diary, but this might have happened only partially. As previously experienced in other trials, many participants reported falls inconsistently. This difficulty of older subjects in recalling is well documented in the literature, turning out to be underestimating single falls and overestimating multiple falls (9, 80). To prevent this bias, completing a daily diary was tested (81); however, people reporting a high number of falls did not return the diary at the end of the trial (82). Different options were proposed in the literature to deal with this issue: monthly diary return through postal service (81), monthly follow-up calls to punctually record falls (83), monetary incentives for monthly diary return (84), and personalization of the latter (84). In this study, a monthly follow-up call was performed to investigate falls recorded in the previous month, but these data did not match those observed at the twelve-month follow-up when diaries were returned. Moreover, several participants did not return the diary or did not fill it in thoroughly. Thus, data recorded during monthly phone calls were used to estimate the fall rate for statistical analysis. Therefore, we cannot exclude that this lack of accuracy in reporting the primary and secondary endpoints may have impacted the results of this trial as a substantial and not emendable bias.

Third, we observed limited participation, a higher drop-out rate, and low adherence to trial post-test assessment in the Control Group, which might have biased the ANCOVA results at post-test. Therefore, future trials should consider offering a placebo treatment to avoid an excessive loss to follow-up in the control group.

Finally, we have to highlight that the chosen study design did not allow us to collect any information on the efficacy of each intervention separately. Regarding this limitation, there are two aspects to be considered. First, we chose interventions whose efficacy as single interventions had already been reported by literature data. Second, the study design was explicitly devised to test the hypothesis that the five interventions could be more effective if administered in combination rather than separately. Given that the results of this study did not confirm our initial hypothesis, it may be worth considering for future studies the adoption of a study design that may facilitate the assessment of the efficacy of each intervention independently where possible.

### Conclusions

This trial attempted to provide a new concept of intervention to reduce falls in a mixed population of older people. The intervention showed the potentiality of improving balance at post-test, leading to a positive trend toward a lower number of falls, lower fall rates in multiple fallers, a lower mean number of falls per participant, and a lower rate of fall-related severe injuries for the intervention group. However, as these differences were not significant, the proposed intervention must be considered ineffective in reducing the number of falls, the falling probability, and the time to the first fall at the twelve-month follow-up in community-dwelling older adults (with or without neurological diseases) at moderate-to-high fall risk.

Unfortunately, other recent RCTs have reached similar conclusions for other interventions (15, 48, 85). Therefore, the temptation would be high to sustain that, as there are no effective interventions, no further efforts should be made to prevent falls and fall-related injuries and improve safe physical mobility in our aging societies. Indeed, as proposed by a recent commentary (86), there is a need for new, better concepts to increase the efficacy of interventions to reduce falls and their consequences. In this respect, the widespread use of ICT solutions could represent an opportunity to be explored. For instance, regarding this trial, the results might have been different if we could have adopted an ICT-based solution to monitor the participants' activity levels and record any eventual falls. In this way, we would have overcome the limitations imposed by the inaccuracy of the fall diaries.

We believe that future studies exploring different effects of combined multiple-component and personalized multifactorial interventions to reduce falls and subsequent consequences should be undertaken with a clear plan for overcoming the limitations highlighted in the PRE.C.I.S.A. study.

## PRECISA study group members

Ospedale Civile di Baggiovara (Modena, Italy);
  - Rehabilitation Medicine: Fabio La Porta, Serena Caselli, Pierina Viviana Clerici, Stefano Cavazza, Valeria Serraglio, Maria Cristina Vannini, Federica Bovolenta, Giada Lullini, Silvia Puglisi, Angela Gallo;
  - Elderly Medicine: Chiara Mussi, Marco Bertolotti, Roberto Scotto, Giulia Lancellotti;
  - Neurology: Franco Valzania, Francesca Falzone;
  - Primary Care Department: Monica Montanari, Maria Luisa De Luca, Emanuela Malagoli, Elisa Franchini;
  - Research and Innovation Unit: Luisa Palmisano, Franca Serafini.

Arcispedale Santa Maria Nuova (Reggio Emilia, Italy);
  - Rehabilitation Medicine: Claudio Tedeschi, Gioacchino Anselmi, Valentina D'Alleva, Mariangela Di Matteo, Rosalinda Ferrari, Stefania Costi, Filomena Simeone, Giulia D'Apote, Alessandra Rizzica, Maria Beatrice Galavotti, Marta Ghirelli;
  - Elderly Medicine: Giulio Pioli, Chiara Bendini, Giulia Lancellotti;
  - Neurology: Massimo Bondavalli, Eleni Georgopoulos

University of Modena and Reggio Emilia, Unit of statistical and methodological support for clinical research;
  - Roberto D'Amico, Sara Balduzzi, Roberto Vicini, Federico Banchelli

University of Bologna, Department of Electrical, Electronic, and Information Engineering “Guglielmo Marconi” (DEI);
  - Lorenzo Chiari, Sabato Mellone, Alice Coni.

## Data availability statement

The raw data supporting the conclusions of this article are available for download at Zenodo.org (according to the license Creative Commons Attribution 4.0 International) from the following link: https://doi.org/10.5281/zenodo.6954088.

## Ethics statement

The studies involving human participants were reviewed and approved by Provincial Ethics Committees of Modena and Reggio Emilia, Italy. The patients/participants provided their written informed consent to participate in this study.

## Author contributions

All authors listed have made a substantial, direct, and intellectual contribution to the work and approved it for publication.

## Funding

This study was financed by the Agenzia Socio-Sanitaria Regionale della Regione Emilia-Romagna (Italy) in the context of the Programma di Ricerca Regione-Università 2013 (PRUA2-2013-00002056).

## Conflict of interest

The authors declare that the research was conducted in the absence of any commercial or financial relationships that could be construed as a potential conflict of interest.

## Publisher's note

All claims expressed in this article are solely those of the authors and do not necessarily represent those of their affiliated organizations, or those of the publisher, the editors and the reviewers. Any product that may be evaluated in this article, or claim that may be made by its manufacturer, is not guaranteed or endorsed by the Publisher.
