# Supplementary material for: Efficacy of a multiple-component and multifactorial personalized fall prevention program in a mixed population of community-dwelling older adults with stroke, Parkinson's Disease, or frailty compared to usual care: The PRE.C.I.S.A. randomized controlled trial
Source: Front Neurol. 2022 Sep 1;13:943918. doi: 10.3389/fneur.2022.943918 (PMC9475118; doi:10.3389/fneur.2022.943918)
Supplement: Supplementary file 1 [file Data_Sheet_1.pdf]

Supplementary Material 1 - Group intervention protocol details

As described in the Methods section, participants in the IG were taken in charge by an interdisciplinary team including a Physiatrist (P), a Physiotherapist (PT), a Geriatrician (G), and a Neurologist (N) who administered synergically the following five interventions.

1. **Group exercise sessions:** one weekly six-person group session of sixty minutes for eleven weeks. Each session was composed of the following parts:

   i. Warming-up (five minutes): head, neck, trunk, and ankle movements, back and knee extensions, walking on the spot;

   ii. Three-station circuit training (thirty-five minutes):

      - Station 1 – Muscular strength exercises: ankle plantarflexion, squat, chair standing, and frontal step (all these exercises were realized with hand support and, when possible, wearing a weight vest).
      - Station 2 – Balance exercises: reaching in standing position, tandem standing, and single-leg standing (1-6 weeks); sidewalk, tandem walk, and toe walk (7-11 weeks).
      - Station 3 – Recovery techniques from falling: seven steps with the 'backward-chaining method', starting from the sitting or standing position(1).

   iii. Dynamic balance and walking, considering the base pathology (ten minutes):

      - Obstacle courses (walking with motor and dual cognitive tasks);
      - Walking exercises (direction and speed changes, associated activities with arms, in crowded contexts; in PD use of visual and auditory cues);
      - Climbing stairs.

   The remaining ten minutes were destined to rest and control the physical activity report diary. During the first session, the Physiotherapist delivered a weight vest to each IG participant and verified the initial level for each of the cited three circuit training stations. Specifically:

   - Station 1 – Muscular strength exercises:
      - First session: for each exercise and each side (in the case of bilateral exercises), the Physiotherapist calculated the subject's maximum weight (1RM) based on the highest number of consecutive repetitions performed by him/her (starting from 1,5 kg, then calculate the 1RM and the percentage of 65% of the 1RM using ad hoc tables built on the average result of three spread formulas (Brzycki, Baechle, Epley)) and noted it in the participant's group treatment chart.

  - Third-Fifth-Seventh-Ninth session: for each exercise and side, the Physiotherapist checked the possible progression (starting from the current working weight, increasing progressively 500 grams to recalculate the 65% of 1RM as described) and noted it in the participant's group treatment chart.

  During the 1RM, the participant had to work to a CR-10 Borg Scale level of '10 – extremely strong', while during muscular strength group exercise with a CR-10 Borg Scale level of '5 – Strong'.

- Station 2 – Balance exercises:
  - First session: for each exercise, the Physiotherapist defined the initial difficulty level (4 levels A-B-C-D of increasing difficulty). He started from medium-high C level, then receded or advanced according to the subject's condition verified during pre-test evaluations.
  - Third-Fifth-Seventh-Ninth session: for each exercise, the Physiotherapist checked the possible progression to the next level, which could occur if the participant realized the activity in a safe condition.
- Station 3 – Recovery techniques from falling:
  - In the first session, the Physiotherapist proposed performing steps 1 and 2, then further 5 steps (to 7), one for each session.
  - It was recommended that the Physiotherapist review the steps of the previous weeks and set the next step for every session.
  - The subject passed to the next step only when he/she could safely perform the previous steps.

At each group session, participants must have their weight vest, their fall-physical activity report diary of the current month, and their manual of the home exercise program (see next point 4).

During rest periods from exercises, participants delivered their fall-physical activity report diary and the completed 'fall report' in case of at least one fall during the week. In addition, at the end of the session, the Physiotherapist updated the manual of the home exercise program with the week-level progression of the exercises (the program changed every two weeks, but depending on individual needs, it was possible to add other series of the same exercise in the intermediate weeks).

2. **Group education sessions on fall risk factors:** one weekly thirty-minute session (held after the group exercise session) for eleven weeks focused on modifiable fall risk factors and risky behaviors. Two parts constituted each session: a ten-minute frontal lesson on a specific theme held by a component of the interdisciplinary team, followed by a twenty-minute group discussion on the lesson content (participants, their caregivers, and the professional). During the eleven weeks, the following topics were proposed:

   i. Why is it important to prevent falls? Why do you fall? (G)

ii. The importance of regular physical exercise (P)
iii. Behaviors at risk of falling (PT)
iv. Drugs and falls (G)
v. Home safety (PT)
vi. Postural hygiene of feet and footwear (PT)
vii. The benefits of a healthy and proper diet and the adoption of healthy lifestyles in fall prevention (G)
viii. Attention and falls (N)
ix. Vision impairment and falls (G)
x. Osteoporosis and falls (G)
xi. Why is it important to continue exercising? (PT)

A handbook summarizing these topics was provided to each participant at the beginning of the first education session.

3. **A personalized plan of reducing domestic fall risk factors following a home visit performed by the Physiotherapist within the first week of treatment.** During this visit, lasting sixty-ninety minutes, the Physiotherapist filled out the 'Home environmental risks questionnaire' and compared it with the same questionnaire compiled by the participant at the pre-test assessment. This questionnaire investigated the following macro-areas related to modifiable risk factors:
   - Slippery floors (outside the house, inside the house);
   - Stairs (handrail, height, tread, scale width);
   - Lighting;
   - Carpets, doormats, movable floor coverings, electric wires;
   - Isolated steps, important disconnections;
   - Furniture and ornaments (chairs, table, protruding feet);
   - Objects at high height;
   - Bed height;
   - Bathroom fixtures (toilet height, shower/bath surfaces).

Then, the Physiotherapist gave specific recommendations with proposals for correcting the detected modifiable risk factors by delivering the 'Suggestions for the reduction of environmental risks at home' sheet in which the actual present hazards were highlighted. The PT also verified the presence of the fall-physical activity report diary, delivered at the time of

recruitment, in a position that facilitated the compilation in case of fall (e.g., hanging on the wall in the living room/ kitchen, etc.).

Finally, during the following three home visits related to the personalized home exercise program, the Physiotherapist checked the implementation of recommended corrections in the first home access and filled the ‘Check-list for reduction of environmental risk factors at home’.

4. **A personalized home exercise program**, coordinated with the group exercise program aimed at improving strength, static and dynamic balance, and acquiring a long-term daily habit of exercise and physical activity in the context of a progressive and permanent adoption of a healthy and active lifestyle. The Physiotherapist devised this program during an initial home visit (2nd week) and subsequently monitored it within two further home visits (4th and 6th weeks).

During the initial home visit, an illustrated manual containing strength and balance exercises was provided and explained to each participant, based on the first group exercise session (in which the subject’s maximum weight (1RM) for strength exercises and the initial difficulty level for balance exercises were calculated). These exercises were chosen between those the subject performed with higher safety in the group session. The indications given to the study’s subjects were:

- To perform muscular strength and balance exercises two more times a week, in addition to the group session conducted at the center, for about thirty minutes, possibly on alternate days to allow rest between one session and the following;
- To perform a thirty-minute walking session on rest days from exercises, at least twice a week. These sessions could be divided into shorter sessions (e.g., at the beginning three ten-minute sessions, alternate with rest, and then increase reaching a single thirty-minute session);
- To register the performed physical activity in their fall-physical activity report diary of the current month, which the Physiotherapist checked during the weekly group session.

During the subsequent two visits, the Physiotherapist verified the setting adequacy and modality in which the participant performed the suggested exercises and updated the fall-physical activity report diary.

Finally, in all the three home access linked to the home exercise program, the Physiotherapist checked the implementation/maintenance of the recommendations on risk factors correction given in the first-week home visit.

5. **A multifactorial personalized intervention aiming at modifying additional risk factors** which were performed by the interdisciplinary team and included the following interventions:

- Review of medications, including psychotropic medications (N and G), antiparkinsonian drugs (N), and cardiovascular medications (G);
- Management of unaddressed visual impairments (G): ophthalmologist referral, lens prescription, suggestions regarding the limitation of bifocal lenses;
- Management of unaddressed cardiovascular issues (G), such as postural hypotension, covert cardiac failure, and abnormalities of cardiac rhythm, eventual cardiology referral;
- Vitamin D prescription (G);
- Improvement of nutritional state (G), with prescription of caloric-proteic integration and/or nutritional referral;
- Management of muscle-skeletal issues, including spasticity (P and PT);
- Education about foot self-care, including podologist referral if appropriate (P);
- Assessment, prescription, and final testing of orthesis and mobility aids, including proper shoes (P and PT).

Interventions one to four were administered to all IG participants (multiple-component intervention), whereas the multifactorial intervention (intervention five) was personalized based on the individual fall risk profile devised on the pre-test assessment. Furthermore, interventions one, two, and five were conducted within an outpatient setting, while interventions three and four were home-based.

References (Supplementary Material 1)

1. Reece AC, Simpson JM. Preparing Older People to Cope after a Fall. *Physiotherapy* (1996) **82**:227–235. doi: 10.1016/S0031-9406(05)66877-0
