# Supplementary material for: Efficacy of a multiple-component and multifactorial personalized fall prevention program in a mixed population of community-dwelling older adults with stroke, Parkinson's Disease, or frailty compared to usual care: The PRE.C.I.S.A. randomized controlled trial
Source: Front Neurol. 2022 Sep 1;13:943918. doi: 10.3389/fneur.2022.943918 (PMC9475118; doi:10.3389/fneur.2022.943918)
Supplement: Supplementary file 2 [file Data_Sheet_2.pdf]

Supplementary Material 2 - Outcome measure details (AAE,OAE, T1 and T3assessments)

Instrument	ICF domain	ICF chapter	Assessed variable	Assessor
30-second Chair Standing test (CS)(1)	Activity and participation	d4 Mobility	Balance (screening)	Physiotherapist (VOE)
Abbreviated Mental Test Score (AMTS)(2)	Body functions	b1 Mental functions	Cognitive functioning (screening)	Physiotherapist (VOE)
Activity-specific Balance Confidence Scale (ABC Scale)(3)	Body functions	b1 Mental functions	Confidence in his/her own balance	Physiatrist (T1 and T3)
Addenbrooke's Cognitive Examination-Revised (ACE-R)(4)	Body functions	b1 Mental functions	Cognitive functioning	Geriatrician (T1 and T3)
Barthel Index (BI)(5)	Activity and participation	d5 Self-care	ADL independence	Physiotherapist (T1 and T3)
Berg Balance Scale (BBS)(6)	Activity and participation	d4 Mobility	Balance	Physiotherapist (T1 and T3)
Body Mass Index (BMI)(7)	Body functions	b5 Functions of the digestive, metabolic and endocrine systems	Body mass	Geriatrician (T1 and T3)
Body weight	Body functions	b5 Functions of the digestive, metabolic and endocrine systems	Body weight	Geriatrician (T1 and T3)
COMPosite Autonomic Symptoms Score (COMPASS-31)(8)	Body functions	b4 Functions of the cardiovascular, haematological, immunological, and respiratory systems	Autonomic symptoms	Geriatrician (T1 and T3)
Cumulative Illness Rating Scale (CIRS)(9)	-	-	Comorbidity profile	Geriatrician (T1 and T3)
Fall Risk Assessment Tool (FRAT)_Stapleton(10)	-	-	Global fall risk	Physiotherapist (VOE)
Falls Efficacy Scale-International (FES-I)(11)	Body functions	b1 Mental functions	Fear of falling	Physiatrist (T1 and T3)
Freezing Of Gait Questionnaire (FOG)(12)	Body functions	b7 Neuromusculoskeletal and movement-related functions	Freezing in people with PD	Neurologist (T1 and T3)
FROP-Com(13) and FROP-Com Screen(14)	-	-	Global fall risk	Interdisciplinary (T1 and T3)
Modified Fugl-Meyer Assessment – Part A, Lower extremity (Lindmark e Hamrin)(15)	Body functions	b7 Neuromusculoskeletal and movement-related functions	Motor and sensitive functioning of lower extremities	Physiotherapist (Pre-test)

Fullerton Advanced Balance Scale (FABS)(16)	Activity and participation	d4 Mobility	Balance	Physiotherapist (T1 and T3)
Functional Reach Test (FRT)(17)	Activity and participation	d4 Mobility	Balance (screening)	Physiotherapist (VOE)
Geriatric Depression Scale (GDS)(18)	Body functions	b1 Mental functions	Mood	Geriatrician (T1 and T3)
Lawton's index (LI)(19)	Activity and participation	d4 Mobility, d6 Domestic life, d8 Major life areas	IADL independence	Geriatrician (T1 and T3)
Mini Balance Evaluation - System Test (Mini-BESTest)(20)	Activity and participation	d4 Mobility	Balance	Physiotherapist (T1 and T3)
Mini Nutritional Assessment (MNA)(21)	Body functions	b5 Functions of the digestive, metabolic and endocrine systems	Nutritional status	Geriatrician (T1 and T3)
Mini-Mental State Examination (MMSE)(22)	Body functions	b1 Mental functions	Cognitive functioning	Geriatrician (T1 and T3)
National Institute of Health Stroke Scale (NIHSS)(23)	-	-	Stroke impairment profile	Neurologist (T1 and T3)
Number of home environment-related risks	Environmental factors	e115 Products and technology for personal use in daily living	Home environment-related risks	Physiotherapist (VOE)
Number of medications	Environmental factors	e110 Products or substances for personal consumption	Medications	Geriatrician (T1 and T3)
Orthostatic Hypotension test	Body functions	b4 Functions of the cardiovascular, haematological, immunological, and respiratory systems	Orthostatic hypotension	Geriatrician (T1 and T3)
Parkinson's Disease Questionnaire-39 (PDQ-39)(24)	-	-	Quality of life	Neurologist (T1 and T3)
Performance-Oriented Mobility Assessment (POMA)(25)	Activity and participation	d4 Mobility	Balance and walking (Tinetti scales)	Physiotherapist (T1 and T3)
Rivermead Mobility Index (RMI)(26)	Activity and participation	d4 Mobility	Mobility (according to the subject)	Physiotherapist (T1 and T3)
Satisfaction With Life Scale (SWLS)(27)	-	-	Quality of life	Physiotherapist (VOE)
Short Physical Performance Battery (SPPB)(28)	Activity and participation	d4 Mobility	Lower extremity strength (screening)|	Physiotherapist (VOE)
Six Minutes Walking Test (6minWT)(29)	Activity and participation	d4 Mobility	Walking: endurance	Physiotherapist (T1 and T3)
Snellen Chart	Body functions	b2 Sensory functions and pain	Visual acuity	Physiotherapist (VOE)

Standing Balance (from 4 Stage Balance Test) (SB)(30)	Activity and participation	d4 Mobility	Balance (screening)	Physiotherapist (VOE)
Stroke Specific Quality of Life scale (SS-QOL)(31)	-	-	Quality of life in people with stroke	Neurologist (T1 and T3)
Ten Meters Walking Test (10mtWT)(32)	Activity and participation	d4 Mobility	Walking: speed	Physiotherapist (VOE)
Timed Up&Go Test (TUG)(33)	Activity and participation	d4 Mobility	Balance (screening)	Physiotherapist (VOE)
Unified Parkinson's Disease Rating Scale (UPDRS I-VI)(34)	-	-	PD functioning profile	Neurologist (T1 and T3)
Visual Analogue Scale (VAS)(35)	Body functions	b2 Sensory functions and pain	Neuromusculoskeletal pain	Physiatrist (T1 and T3)
Walking Handicap Scale (Perry&Garret) (WHS)(36)	Activity and participation	d4 Mobility	Walking independence	Physiatrist (T1 and T3)
WHO Rose questionnaire(37)	Body functions	b4 Functions of the cardiovascular, haematological, immunological, and respiratory systems	Claudicatio intermittens	Geriatrician (T1 and T3)
Word Health Organization Disability Assessment Schedule 2.0 - 12 item version (WHODAS 2.0)(38)	Activity and participation	d4 Mobility, d5 Self care, d6 Domestic life, d7 Interpersonal interactions, and relationships	Social participation	Physiatrist (T1 and T3)
World Health Organization Quality of Life – Older adults (WHOQoL-OLD)(39)	-	-	Quality of life in older people	Geriatrician (T1 and T3)

References (Supplementary Material 2)

1. Jones CJ, Rikli RE, Beam WC. A 30-s chair-stand test as a measure of lower body strength in community-residing older adults. *Res Q Exerc Sport* (1999) 70:113–119. doi: 10.1080/02701367.1999.10608028
2. Hodkinson HM. Evaluation of a mental test score for assessment of mental impairment in the elderly. *Age Ageing* (1972) 1:233–238. doi: 10.1093/ageing/1.4.233
3. Powell LE, Myers AM. The Activities-specific Balance Confidence (ABC) Scale. *J Gerontol A Biol Sci Med Sci* (1995) 50A:M28-34. doi: 10.1093/gerona/50a.1.m28
4. Mioshi E, Dawson K, Mitchell J, Arnold R, Hodges JR. The Addenbrooke's Cognitive Examination Revised (ACE-R): a brief cognitive test battery for dementia screening. *Int J Geriatr Psychiatry* (2006) 21:1078–1085. doi: 10.1002/gps.1610
5. Mahoney FI, Barthel DW. Functional evaluation: the Barthel Index. *Md State Med J* (1965) 14:61–65.
6. Berg KO, Wood-Dauphinee SL, Williams JI, Maki B. Measuring balance in the elderly: validation of an instrument. *Can J Public Health Rev*

Can Sante Publique (1992) 83 Suppl 2:S7-11.
7. Eknoyan G. Adolphe Quetelet (1796 1874) the average man and indices of obesity. *Nephrol Dial Transplant* (2007) 23:47–51. doi: 10.1093/ndt/gfm517
8. Sletten DM, Suarez GA, Low PA, Mandrekar J, Singer W. COMPASS 31: a refined and abbreviated Composite Autonomic Symptom Score. *Mayo Clin Proc* (2012) 87:1196–1201. doi: 10.1016/j.mayocp.2012.10.013
9. Linn BS, Linn MW, Gurel L. Cumulative Illness Rating Scale. *J Am Geriatr Soc* (1968) 16:622–626. doi: 10.1111/j.1532-5415.1968.tb02103.x
10. Stapleton C, Hough P, Oldmeadow L, Bull K, Hill K, Greenwood K. Four-item fall risk screening tool for subacute and residential aged care: The first step in fall prevention. *Australas J Ageing* (2009) 28:139–143. doi: 10.1111/j.1741-6612.2009.00375.x
11. Yardley L, Beyer N, Hauer K, Kempen G, Piot-Ziegler C, Todd C. Development and initial validation of the Falls Efficacy Scale-International (FES-I). *Age Ageing* (2005) 34:614–619. doi: 10.1093/ageing/afi196
12. Giladi N, Shabtai H, Simon ES, Biran S, Tal J, Korczyn AD. Construction of freezing of gait questionnaire for patients with Parkinsonism. *Parkinsonism Relat Disord* (2000) 6:165–170. doi: 10.1016/S1353-8020(99)00062-0
13. Russell MA, Hill KD, Blackberry I, Day LM, Dharmage SC. The reliability and predictive accuracy of the falls risk for older people in the community assessment (FROP-Com) tool. *Age Ageing* (2008) 37:634–639. doi: 10.1093/ageing/afn129
14. Russell MA, Hill KD, Day LM, Blackberry I, Gurrin LC, Dharmage SC. Development of the Falls Risk for Older People in the Community (FROP-Com) screening tool. *Age Ageing* (2009) 38:40–46. doi: 10.1093/ageing/afn196
15. Lindmark B, Hamrin E. Evaluation of functional capacity after stroke as a basis for active intervention. Presentation of a modified chart for motor capacity assessment and its reliability. *Scand J Rehabil Med* (1988) 20:103–109.
16. Rose DJ, Lucchese N, Wiersma LD. Development of a multidimensional balance scale for use with functionally independent older adults. *Arch Phys Med Rehabil* (2006) 87:1478–1485. doi: 10.1016/j.apmr.2006.07.263
17. Duncan PW, Weiner DK, Chandler J, Studenski S. Functional reach: a new clinical measure of balance. *J Gerontol* (1990) 45:M192-197. doi: 10.1093/geronj/45.6.m192
18. Hoyl MT, Alessi CA, Harker JO, Josephson KR, Pietruszka FM, Koelfgen M, Mervis JR, Fitten LJ, Rubenstein LZ. Development and testing of a five-item version of the Geriatric Depression Scale. *J Am Geriatr Soc* (1999) 47:873–878. doi: 10.1111/j.1532-5415.1999.tb03848.x
19. Lawton MP, Brody EM. Assessment of older people: self-maintaining and instrumental activities of daily living. *The Gerontologist* (1969) 9:179–186.
20. Franchignoni F, Horak F, Godi M, Nardone A, Giordano A. Using psychometric techniques to improve the Balance Evaluation Systems Test: the mini-BESTest. *J Rehabil Med* (2010) 42:323–331. doi: 10.2340/16501977-0537
21. Vellas B, Villars H, Abellan G, Soto ME, Rolland Y, Guigoz Y, Morley JE, Chumlea W, Salva A, Rubenstein LZ, et al. Overview of the MNA--Its history and challenges. *J Nutr Health Aging* (2006) 10:456–463; discussion 463-465.
22. Folstein MF, Folstein SE, McHugh PR. “Mini-mental state”. A practical method for grading the cognitive state of patients for the clinician. *J Psychiatr Res* (1975) 12:189–198. doi: 10.1016/0022-3956(75)90026-6
23. Brott T, Adams HP, Olinger CP, Marler JR, Barsan WG, Biller J, Spilker J, Holleran R, Eberle R, Hertzberg V. Measurements of acute cerebral infarction: a clinical examination scale. *Stroke* (1989) 20:864–870. doi: 10.1161/01.str.20.7.864
24. Peto V, Jenkinson C, Fitzpatrick R. PDQ-39: a review of the development, validation and application of a Parkinson’s disease quality of life questionnaire and its associated measures. *J Neurol* (1998) 245 Suppl 1:S10-14. doi: 10.1007/pl00007730
25. Tinetti ME, Williams TF, Mayewski R. Fall risk index for elderly patients based on number of chronic disabilities. *Am J Med* (1986) 80:429–

434. doi: 10.1016/0002-9343(86)90717-5
26. Collen FM, Wade DT, Robb GF, Bradshaw CM. The Rivermead Mobility Index: a further development of the Rivermead Motor Assessment. *Int Disabil Stud* (1991) 13:50–54. doi: 10.3109/03790799109166684
27. Diener E, Emmons RA, Larsen RJ, Griffin S. The Satisfaction With Life Scale. *J Pers Assess* (1985) 49:71–75. doi: 10.1207/s15327752jpa4901_13
28. Guralnik JM, Simonsick EM, Ferrucci L, Glynn RJ, Berkman LF, Blazer DG, Scherr PA, Wallace RB. A short physical performance battery assessing lower extremity function: association with self-reported disability and prediction of mortality and nursing home admission. *J Gerontol* (1994) 49:M85-94. doi: 10.1093/geronj/49.2.m85
29. ATS Committee on Proficiency Standards for Clinical Pulmonary Function Laboratories. ATS statement: guidelines for the six-minute walk test. *Am J Respir Crit Care Med* (2002) 166:111–117. doi: 10.1164/ajrccm.166.1.at1102
30. Rossiter-Fornoff JE, Wolf SL, Wolfson LI, Buchner DM. A cross-sectional validation study of the FICSIT common data base static balance measures. Frailty and Injuries: Cooperative Studies of Intervention Techniques. *J Gerontol A Biol Sci Med Sci* (1995) 50:M291-297. doi: 10.1093/gerona/50a.6.m291
31. Williams LS, Weinberger M, Harris LE, Clark DO, Biller J. Development of a stroke-specific quality of life scale. *Stroke* (1999) 30:1362–1369. doi: 10.1161/01.str.30.7.1362
32. Collen FM, Wade DT, Bradshaw CM. Mobility after stroke: reliability of measures of impairment and disability. *Int Disabil Stud* (1990) 12:6–9. doi: 10.3109/03790799009166594
33. Podsiadlo D, Richardson S. The timed “Up & Go”: a test of basic functional mobility for frail elderly persons. *J Am Geriatr Soc* (1991) 39:142–148. doi: 10.1111/j.1532-5415.1991.tb01616.x
34. Fahn S, Elton R. “Members of the UPDRS Development Committee.,” *Recent Developments in Parkinson’s Disease*. Florham Park, NJ. p. pp 15 3-163, 293-304
35. Gould D. Visual Analogue Scale (VAS). *J Clin Nurs* (2001)697–706.
36. Perry J, Garrett M, Gronley JK, Mulroy SJ. Classification of walking handicap in the stroke population. *Stroke* (1995) 26:982–989. doi: 10.1161/01.str.26.6.982
37. Rose GA. The diagnosis of ischaemic heart pain and intermittent claudication in field surveys. *Bull World Health Organ* (1962) 27:645–658.
38. Ustün TB, Chatterji S, Kostanjsek N, Rehm J, Kennedy C, Epping-Jordan J, Saxena S, von Korff M, Pull C, WHO/NIH Joint Project. Developing the World Health Organization Disability Assessment Schedule 2.0. *Bull World Health Organ* (2010) 88:815–823. doi: 10.2471/BLT.09.067231
39. Power M, Quinn K, Schmidt S, WHOQOL-OLD Group. Development of the WHOQOL-old module. *Qual Life Res Int J Qual Life Asp Treat Care Rehabil* (2005) 14:2197–2214. doi: 10.1007/s11136-005-7380-9
